# Design, Synthesis and Biological Evaluation of (2′,5′ and 3′5′-Linked) cGAMP Analogs that Activate Stimulator of Interferon Genes (STING)

**DOI:** 10.3390/molecules25225285

**Published:** 2020-11-12

**Authors:** Xin Xie, Junyi Liu, Xiaowei Wang

**Affiliations:** 1 Department of Chemical Biology, School of Pharmaceutical Sciences, Peking University, Beijing 100191, China; chemsp621@bjmu.edu.cn; 2 State Key Laboratory of Natural and Biomimetic Drugs, Peking University, Beijing 100191, China

**Keywords:** STING agonist, cyclic dinucleotides (CDNs), SPR, immunotherapy

## Abstract

Stimulator of interferon genes (STING) is an endoplasmic reticulum adaptor transmembrane protein that plays a pivotal role in innate immune system. STING agonists, such as endogenous cyclic dinucleotide (CDN) cyclic GMP-AMP (cGAMP), have been used in diverse clinical research for immunogenic tumor clearance, antiviral treatments and vaccine adjuvants. CDNs containing noncanonical mixed 3′-5′ and 2′-5′ phosphodiester linkages show higher potency in the activation of the STING pathway. In this study, a series of 2′3′-CDNs were designed and synthesized through a modified one-pot strategy. We then established a surface plasmon resonance (SPR)-based binding assay to quantify the binding affinities of synthesized CDNs for human STING, which requested a minuscule amount of sample without any pretreatment. Using this assay, we identified compound **8d** (K_D_ = 0.038 μM), a novel CDN that showed higher binding affinity with hSTING than cGAMP (K_D_ = 0.543 μM). Cellular assays confirmed that **8d** could trigger the expression of type I IFNs and other proinflammatory cytokines more robust than cGAMP. **8d** also exhibited more resistant than cGAMP to enzymatic cleavage in vitro, indicating the successful improvement in drug availability. These findings provide guidelines for the design and structural optimization of CDNs as STING agonists.

## 1. Introduction

Stimulator of interferon genes (STING) is an endoplasmic reticulum adaptor protein with four transmembrane domains [1], and it is widely expressed in immune cells. Once double-stranded DNA (dsDNA) or single-stranded DNA (ssDNA) leakage from the nucleus or induced by viruses or bacteria are sensed by cGAS in the cytoplasm, endogenous second messenger 2′3′ cyclic GMP-AMP (cGAMP) will be synthesized [2,3]. STING can be directly activated by cGAMP and the other cyclic dinucleotides (CDNs), such as c-di-AMP and c-di-GMP [4].

Following stimulation by cGAMP, the activated STING dimers will translocate from the endoplasmic reticulum (ER) to the Golgi apparatus [5], and then, the downstream kinase TANK-binding kinase 1 (TBK1) are recruited to the C-terminal tail of STING, which results in TBK1 autophosphorylation [6,7] and the subsequent phosphorylation of interferon regulatory factor 3 (IRF3) [8,9]. The phosphorylated IRF3 dimers ultimately enter the nucleus and upregulate the expression of type I IFNs and inflammatory cytokine genes [7].

The function of the STING-TBK1-IRF3 pathway has been extensively studied in multiple infectious disease models, and the engagement of STING has been demonstrated indispensable for host immune defense against microbes, DNA viruses and retroviruses, such as HIV and SIV [10,11].

This signaling pathway also has been demonstrated to play a pivotal role in the antitumor immune response, and numerous studies have suggested that the activation of STING present great potential for the treatment of cancer. The intratumoral administration of natural CDNs STING agonists contributes to tumor volume regression and significantly increase the survival rate in various cancer models in mice [12,13,14]. Moreover, STING activations by intratumoral injection of CDNs can effectively reinforce the antitumor potency and reduce the negative impact of radiotherapy or chemotherapy and synergize with checkpoint inhibitors [15,16,17]. Defined innate immune mechanisms reveal that STING agonists possess great power in antiviral processes, and antitumor immune responses are mainly attributed to the secretion of type I IFN and a broad chemokine profile, such as CXCL10 and IL6, which facilitate the recruitment and activation of natural killer (NK) cells and T cells in the tumor microenvironment [18,19,20]. Additionally, cGAMP and other CDN STING agonists have been shown to be potential vaccine adjuvants that boost the generation of pathogen-specific antibodies and strengthen T-cell responses in mice models [21,22].

**Figure 1:**
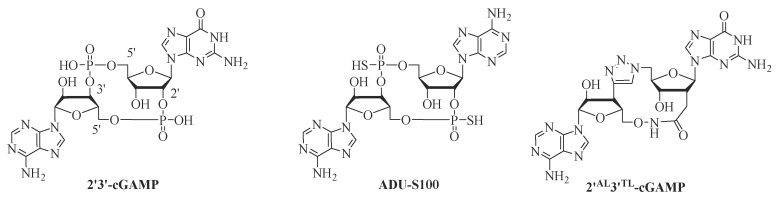
Structures of 2′3′-cyclic GMP-AMP (cGAMP), ADU-S100 and 2′^AL^3′^TL^-cGAMP.

Thus, the pharmacologic-targeted STING-dependent signaling pathway shows promise in exploiting novel therapeutic approaches for diverse clinically applications. The natural CDNs such as c-di-GMP, c-di-AMP can directly bind to STING. CDNs, consisting of two nucleotides cyclized by two 3′5′-phosphodiester bonds, were first discovered in bacteria and cyclized by a 2′5′-phosphodiester bond, and a 3′5′-phosphodiester bond named 2′3′-cGAMP (Figure 1) is the only known mammalian CDN. Interestingly, contrasted to other natural CDNs, 2′3′-cGAMP shows an extremely higher affinity and elicits more drastic immune responses. Another 2′3′-CDN, ADU-S100, with thiophosphatediester bonds, has escalated in Phase II for the treatment of cancer. 2′^AL^3′^TL^-cGAMP, another cGAMP analog with one amide and one triazole linkage, was also synthesized to feature better cellular penetrability [23]. Meanwhile, the biocatalytic synthesis of cyclic dinucleotide derivatives by human cGAS was also established. Large collections of modified 2′3′CDNs analogs were enzymatically prepared from the commercially available ATP and GTP analogs. Most substrate derivatives with modifications at the nucleobase, ribose and the α-thiophosphate were accepted [24,25].

Herein, we report the design, synthesis and biological screening of novel CDN-targeted STING. Unlike most of the reported CDNs, whose bases in the constituent nucleosides are two purines, the CDNs we describe here contain one purine and one pyrimidine base. Taking 2′3′-cGAMP as the lead compound, we designed and synthesized eight CDNs that kept the 2′,3′-internucleotide linkage position but varied by one base and phosphate modification (bis-phosphodiester or bis-phosphorothioate). We detected all eight analogs for the binding affinity towards human STING using a surface plasmon resonance (SPR)-based assay. Then, we screened these analogs for the ex vivo production of IFN-β in human leukemic monocytes THP-1 by enzyme-linked immunosorbent assay (ELISA). The most promising analog, **8d**, was further evaluated for the ability to serve in other cytokine productions by RT-qPCR. We also explored the resistance of these compounds to cleavage by serum and nuclease P1 (NP1). We summarize our findings and discuss the structure-activity relationship observations that provided a theoretical basis for structural transformation as a guidance for STING-targeted immunotherapy CDN drug development.

## 2. Results

### 2.1. Design and Synthesis of 2′3′-cGAMP Analogs

**Figure 2:**
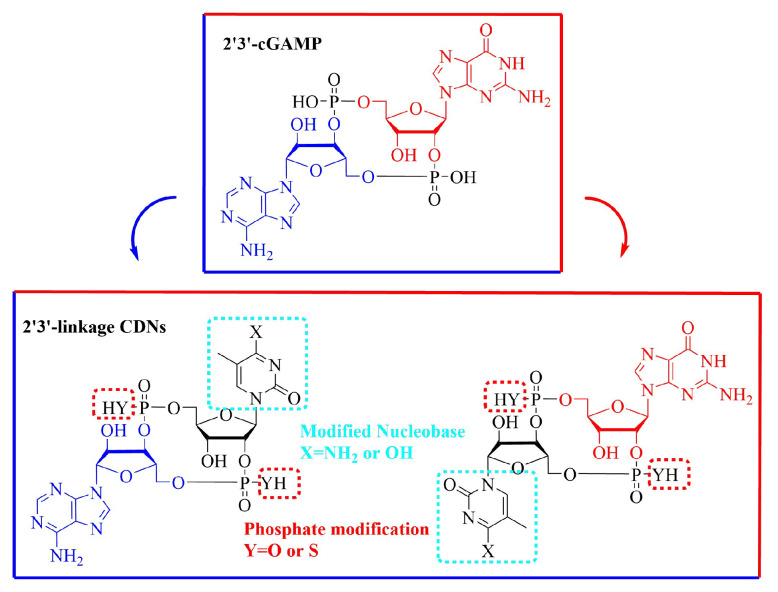
General structure of the cGAMP analogs in the present study.

We began our study by analyzing the interactions between 2′3′-cGAMP and hSTING in the crystal complex (Protein Data Bank (PDB): 4LOH). The two purine moieties, guanosine and adenosine, form hydrogen bonding with STING. Structural modifications launched by replacing the purine by structural similarities are theoretically feasible. In this study, we took conversion of noncanonical nucleosides to CDNs. On the other hand, due to the compelling bioactivities of the 2′3′-phosphodiester-linked CDNs, such as ADU-S100, we decided to focus our efforts on 2′3′-CDNs. To ascertain how these bisphosphorothioate linkages influence the activity of such compounds, the synthesized analogs were systemically designed as a series of bisphosphorothioate and bisphosphodiester-coupled CDNs. Above all, at the onset of this study, we designed a series of 2′3′-CDNs, taking 2′3′-cGAMP as the lead molecule (Figure 2).

**Scheme 1:**
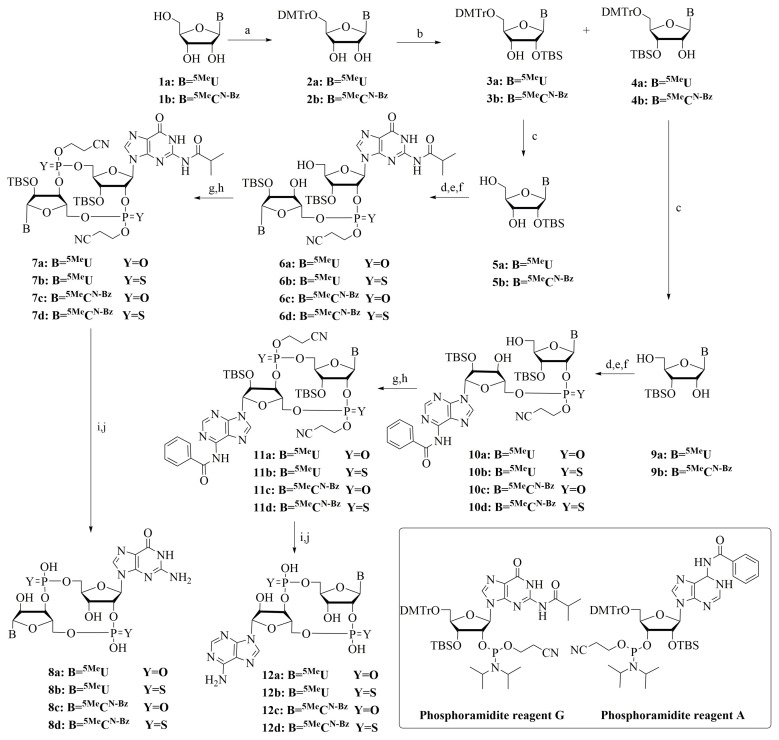
Preparation of 2′3′-cGAMP analogs. Reagents and conditions: (**a**) DMTrCl, pyridinium, rt; (**b**) TBSCl, imidazole, rt; (**c**) dichloroacetic acid, dichloromethane (DCM); (**d**) phosphoramidite reagent G/A, 1*H*-tetrazole, CH_3_CN (**e**) 5.5 M tert-butyl hydroperoxide (tBuOOH) in decane for Y=O or phenylacetyl disulfide (PADS) for Y=S; (**f**) dichloroacetic acid, dichloromethane; (**g**) 2-cyanoethyl- N,N,N’,N’-tetraisopropyl phosphorodiamidite, 1*H*-tetrazole, CAN; (**h**) 5.5 M tert-butyl hydroperoxide (tBuOOH) in decane for Y=O or phenylacetyl disulfide (PADS) for Y=S; (**i**) 33% methylamine (MeNH_2_) in ethanol and (**j**) Et_3_N·3HF in Et_3_N.

We started by preparing 2′ or 3′-protected noncanonical nucleosides (Scheme 1. **5a**, **5b**, **9a** and **9b**), coupled then with the commercially available phosporamidites (Scheme 1) in anhydrous CAN solution. The phosphite products were oxidized with either tert-butyl hydroperoxide (tBuOOH) or sulfurized phenylacetyl disulfide (PADS). The 5′-DMTr-protected group in each compound was removed by dichloroacetic acid to give the linear dimer (**6a**-**6d**, **11a**-**11d**). Then the dimers were directly cyclized in a high-dilution mixture of 2-cyanoethyl-N,N,N’,N’-tetraisopropyl phosphorodiamidite in a dry CAN-pyridine mixture and then treated with either tBuOOH or PADS to provide CDNs with protected groups. These CDNs were deprotected by successive treatment with methylamine and Et_3_N^.^3HF. Due to the high polarity, crude CDNs samples were purified by preparative HPLC. Eight 2′3′-cGAMP analogs were successfully synthesized and characterized.

### 2.2. hSTING-Binding Assays

Direct measurement of affinity constants provide assistance in pharmacokinetic and pharmacodynamic studies. In this study, we evaluated the binding affinities between human STING and the synthesized CNDs by the surface plasmon resonance (SPR)-based assay, which loaded minuscule amounts of STING protein on a biosensor chip, and free-labeled samples were directly detected in short measurement times.

hSTING was firstly immobilized on a CM5 sensor chip via a N-terminal cysteine thiol group, without altering conformation or binding activity. Sufficient STING presented to CDN molecules in the mobile phase. Kinetic parameters generally give both an aggregation constant (ka) and a disaggregation constant (k_d_), from which the equilibrium dissociation constant K_D_ can be calculated by K_D_ = K_d_/K_a_.

Regarding the synthesized CDNs, the dissociation parameters for these complexes are listed in Table 1, and the natural cGAMP bound to STING with a K_D_ of 0.543 μM. The analogs contained a guanosine moiety that exhibited increased binding affinity by about an order of magnitude for hSTING compared to CDNs containing an adenosine moiety. In addition, compounds (**8b**, **8d**, **12b** and **12d**) that contained phosphorothioate generally exhibited higher affinity than their parent phosphodiesters (**8a**, **8c**, **12a** and **12c**). Compound **8d** (K_D_ = 0.038 μM) contained both guanosine moieties, and phosphorothioate linkages exhibited the best binding affinity. In other words, the substitution of O by S directly affected the binding affinity between CDN and STING. This is powerful evidence to confirm that the phosphorothioate or phosphodiester group is a junction site towards STING.

### 2.3. Cytokine Induction In Vitro

**Figure 3:**
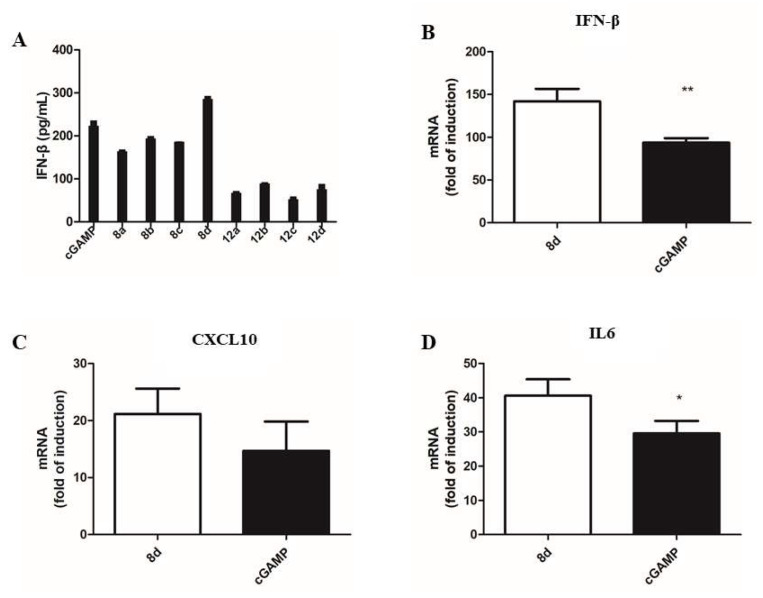
Cytokine induction in THP-1 cells. (**A**) THP-1 cells collected at 18h after cGAMP analogs (1μM) administration were used to detect the production of IFN-β by enzyme-linked immunosorbent assay (ELISA). Expression analysis of IFN-β (**B**), CXCL10 (**C**) and IL6 (**D**) in THP-1 cells after the treatment of **8d** and cGAMP (5 μM) with 12 h was detected by RT-qPCR expressed as a fold induction. Data represent means ± SEM; ** *p* < 0.01 and * *p* < 0.05.

Activation of the STING pathway by the stimulation of CDNs contribute notably to the production of type I interferons. Thus, we evaluated the bioactivity of the synthesized CDNs in THP-1 cells and used IFN-β protein concentrations in cytochylema as a pharmacodynamic marker. These newly synthetized CDNs, whose structures contained a guanosine moiety, showed equivalent or superior activity relative to cGAMP (Figure 3A). A noticeable difference was observed between thiophosphate and phosphodiester linkage analogs. Consistent with the binding affinities, all the phosphorothioate analogs (**8b, 8d, 12b** and **12d**) induced THP-1 cells to produce more IFN-β than the corresponding phosphodiester analogs (**8a, 8c, 12a** and **12c**).

The most promising analog, **8d**, was further evaluated by the function served in immune and inflammatory cytokine responses. We delivered **8d** and cGAMP into human THP-1 cells and examined the mRNA level of IFN-β, CXCL10 and IL6 by RT-qPCR. Consistent with previous studies, STING modulated a robust and sensitive response to cGAMP. What is more, the transcript level of INF-β, proinflammatory cytokines IL-6 and the chemokine CXCL10 were markedly more induced after the treatment with **8d** relative to the levels in the cGAMP-treated group (Figure 3).

### 2.4. Enzymatic Stability in Serum and Nuclease P1

CDNs containing internucleotide phosphodiester bonds can be rapidly hydrolyzed by enzymes in the serum and cytoplasm. As reported, hydrolases such as nuclease P1 (NP1) were proven to cleave CDNs at their phosphodiester linkages. To evaluate the enzymatic stability of the cGAMP analogs, we incubated each analog for 2 h with either NP1 or the serum.

**Figure 4:**
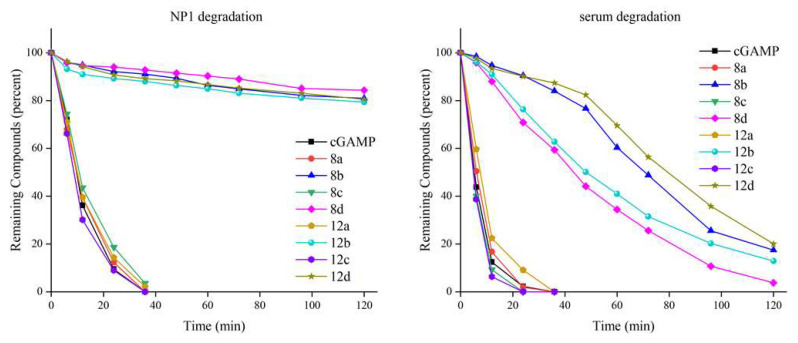
Enzymatic cleavage resistance assays. The cGAMP analogs were incubated with nuclease P1 or serum for 2h; the remaining amount was determined by HPLC and calculated by the peak area.

Monitoring the reaction by HPLC, CDNs that contained phosphodiester linkages were cleaved within 1h, exhibiting half-lives (T_1/2_) of 7–10 min for NP1 and 2–4 min for the serum (Figure 4). No significant difference caused by the base transformation was observed. In contrast, the half-lives of CDNs contained phosphorothioate linkages prolonged both in the serum and NP1. The chemical modification of CDNs by replacing phosphodiester linkages with phosphorothioate linkages allowed more favorable enzymatic stability.

### 2.5. The Result of Molecular Docking

**Figure 5:**
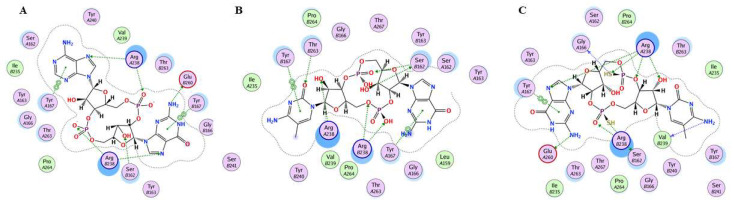
(**A**) The binding mode of cGAMP and hSTING (Protein Data Bank (PDB) code: 4LOH). (**B**)The 2D-binding mode of **8c** and hSTING by docking calculation. (**C**) The 2D-binding mode of **8d** and hSTING by docking calculation.

The docking scores are shown in Table 2. The more negative the docking score, the better the binding of the ligand with the protein. The binding of **8d** with hSTING was better than that of cGAMP and **8c**. The binding modes of **8c** and **8d** with the protein hSTING are illustrated in Figure 5. **8c** and **8d** formed a suitable steric complementarity with the binding site of hSTING. Besides, the hydrogen bond and Π-Π stacking interactions were formed between the molecules and hSTING. The sulfur atom in the phosphorothioate group of **8d**, regarded as a hydrogen bond donor, formed a hydrogen bond with the backbone oxygen atom of Gly166 in subunit A and the side-chain oxygen atom of Ser162 in subunit B, respectively. Van der Waals interactions were formed among **8d** and the surrounding residues. These interactions mainly contributed to the binding energy between **8d** with the protein hSTING. A similar interaction between **8c** and hSTING was found.

## 3. Discussion

As a potential immunotherapy target, STING has attracted considerable attention from academic and commercial researchers. Synthetic STING agonists, especially CDNs, with favorable druglike properties, are currently being developed as adjuvants, antitumor agents and other immunotherapy agents.

Herein, a series of 2′3′-cGAMP analogs containing one purine base and one pyrimidine base were synthesized. Subsequently, SPR was selected to test the binding affinity between synthesized CDNs for its low sample consumption, free solution and short detection time, without any pretreatment in ligand or receptor. The result showed that phosphorothioate analogs exhibited distinct activities relative to their parent (phosphodiester) compounds. Furthermore, phosphorothioate CDNs were also more stable to enzymatic resistance than their corresponding phosphodiester CDNs in vitro. Thus, we speculate that both the binding ability and stability contribute to the superior in vitro of the phosphorothioates relative to the phosphodiesters.

Regarding the structural transformation on the base, we replaced one purine by pyrimidine in cGAMP. In this study, the compounds containing a guanine base (**8a**-**8d**) were slightly more active than compounds containing an adenine base, respectively. To a certain extent, changes in the base group size did not dramatically affect the binding ability and bioactivity of these compounds.

Our screening results demonstrated that the replacement of oxygen atoms by sulfur atoms can upgrade the ligand-STING interactions, which suggests that phosphodiester is an active binding site. The following molecular docking confirmed our hypothesis. These findings provide guidelines for CDN designed targeted STING.

We also observed that **8d** exhibited more affiliative to STING than cGAMP. A further biological activity assay confirmed **8d** as a more active STING agonist. **8d** may have potential and valuable therapeutic applications as aptamers, antisense agents and desirable adjuvant candidates for improving immunotherapy potency against cancer, infectious diseases and other disorders. Large-scale drug modification and STING agonists screening are on the way.

## 4. Materials and Methods

### 4.1. Synthesis and Characterization

All chemicals were purchased from commercial sources and used without further purification, unless otherwise noted. Reactions were usually under an argon atmosphere. ^1^H-, ^13^C- and ^31^P-NMR spectra were recorded on a Bruker spectrometer (Avance III TM 400) (Bruker Biospin, Fällanden, Switzerland) at room temperature with DMSO-d6 or D_2_O as solvent and calibrated with residual undeuterated solvent (DMSO-d6: δH = 2.50 ppm, δC = 39.52 ppm; D2O: δH = 4.79 ppm) as an internal reference (Figures S1–S18). The following abbreviations were used to designate the multiplicities: s = singlet, d = doublet, dd = double doublets and m = multiplet. Mass spectra were recorded on the MDS SCIEX QSTAR system (MDS SCIEX, Vaughan, Canada). All test compounds were assessed for their purity by the Waters 2695 Separations Module HPLC system (Waters, Massachusetts. America) equipped with a PDA W2998 detector with a Waters SymmetryShieldTM C18 column (4.6 mm × 250 mm, 5 μm) using gradients of 5-mM aq triethylammonium sodium bicarbonate and acetonitrile at a flow rate of 0.8 mL/min. The detection wavelength was 254 nm. Purity for all final compounds was confirmed to be greater than 95%.

**5′-*O*-DMTr-5-metheyluridine (2a).** To a solution of 5-metheyluridine, **1a** (12.91 g, 50 mmol) in pyridine (300 mL) was added DMTrCl (23.73 g, 70 mmol). The mixture was stirred at room temperature for 6h, diluted with CH_2_Cl_2_ (400 mL) and washed with sat NaHCO_3_ (2 × 500 mL) and brine (500 mL). The organic layer was dried over Na_2_SO_4_, filtered and concentrated under reduced pressure. The residue was purified by the chromatography on silica gel (DCM: Methanol = 200:1–40:1) to give **2a** as a white foam solid (21.58 g, 77%). ^1^H NMR (400 MHz, DMSO-*d6*) *δ*: 11.37 (s, 1H), 7.51 (s, 1H), 7.39 (d, 2H, *J* = 7.2 Hz), 7.32 (t, 2H, *J* = 8.0, 7.6 Hz), 7.25 (m, 5H, *J* = 8.8, 7.2 Hz), 6.90 (d, 4H, *J* = 8.8 Hz), 5.80 (d, 1H, *J* = 5.2 Hz), 5.47 (d, 1H *J* = 5.6 Hz), 5.17 (d, 1H, *J* = 5.6 Hz), 4.18 (m, 1H, *J* = 5.6, 5.2 Hz), 4.11 (m, 1H, *J* = 5.2, 4.8 Hz), 3.96 (dd, 1H, *J* = 4.0, 2.8 Hz), 3.74 (s, 6H), 3.17–3.25 (m, 2H), 1.42 (s, 3H). ^13^C NMR (101 MHz, DMSO-*d6*) *δ*: 164.3, 158.5, 151.1, 145.2, 136.3, 135.9, 135.7, 130.2, 128.4, 128.1, 127.3, 113.9, 109.8, 88.2, 86.3, 83.0, 73.7, 70.6, 63.9, 55.2, 11,9. MS (ESI) calculated for C_31_H_32_N_2_O_8_: 560.22. found (M + Na)^+^: 583.59.

**5′-*O*-DMTr-5-methyl-N^4^-benzoylcytidine (2b).** Compound **1b** was subjected to a similar procedure to that described for preparation of **2a** to give **2b** (22.57 g; 68%). ^1^H NMR (400 MHz, DMSO-*d6*) *δ*: 13.00 (s, 1H), 8.21 (d, 2H, *J* = 7.2), 7.84 (s, 1H), 7.63 (t, 1H, *J* = 7.6, 7.2 Hz), 7.54 (t, 2H, *J* = 7.6, 7.2 Hz), 7.45 (d, 2H, *J* = 7.6 Hz), 7.29–7.39 (m, 7H), 6.96 (d, 4H, *J* = 8.0 Hz), 5.86 (d, 1H, *J* = 3.6 Hz), 5.26 (d, 1H, *J* = 5.6 Hz), 4.20–4.25 (m, 2H), 4.08 (1H), 3.78 (s, 6H), 3.29–3.33 (m, 2H), 1.64 (s, 3H). ^13^C NMR (101 MHz, DMSO-*d6*) *δ*: 158.4, 145.1, 135.8, 135.6, 133.0, 130.2, 128.8, 128.5, 128.2, 127.3, 113.8, 86.4, 83.4, 74.2, 70.1, 63.5, 60.2, 55.5, 14.4. MS (ESI) calculated for C_38_H_37_N_3_O_8_: 663.26. found ( M+H)^+^: 664.12.

To a solution of **2a** (2.80 g, 5 mmol) in THF (50nL) and pyridine (5 mL) were added imidazole (0.68 g, 10 mmol) and TBSCl (0.90g, 6 mmol). The mixture was stirred at r.t. for 4h, diluted with brine (200 mL) and extracted with EtOAc (3 × 200 mL). The combined organic extracts were dried over anhydrous Na_2_SO_4_ and concentrated under reduced pressure. The crude product was purified by SiO_2_ gel chromatography to afford **3a** (1.82 g, 54%) and **4a** (1.38 g, 41%).

**5′-*O*-DMTr-2′-*O*-TBDMS-5-metheyluridine (3a).**^1^H NMR (400 MHz, CDCl_3_) *δ*: 8.37 (s, 1H), 7.62 (s, 1H), 7.41 (d, 2H, *J* = 7.6 Hz), 7.25–7.34 (m, 7H), 6.86 (d, 4H, *J* = 8.4 Hz), 6.01 (d, 1H, *J* = 5.6 Hz), 4.40 (t, 1H, *J* = 5.6 Hz), 4.30 (m, 1H, *J* = 5.6 Hz), 4.08 (m, 1H), 3.81 (s, 6H), 3.55 (m, 1H), 3.27 (m, 1H), 2.87 (d, 1H), 1.50 (s, 3H), 0.89 (s, 9H), 0.09 (s, 3H), −0.01(s, 3H). ^13^C NMR (101 MHz, CDCl_3_) *δ*: 163.3, 158.8, 150.4, 143.8, 135.7, 135.1, 129.9, 128.1, 127.3 113.4, 111.2, 89.1, 87.3, 84.6, 74.8, 71.8, 62.7, 55.1, 25.5, 17.9, 11.8, −5.0. MS (ESI) calculated for C_37_H_46_N_2_O_8_Si: 674.30. found (M+Na)^+^: 697.74.

**5′-*O*-DMTr-3′-*O*-TBDMS-5-metheyluridine (4a).**^1^H NMR (400 MHz, CDCl_3_) *δ*: 8.30 (s, 1H), 7.68 (s, 1H), 7.41 (d, 2H, *J* = 8.4 Hz), 7.26–7.43 (m, 7H), 6.85 (d, 4H, *J* = 8.8 Hz), 6.06 (d, 1H, *J* = 5.6 Hz), 4.51 (t, 1H, *J* = 5.2 Hz), 4.32 (m, 1H, *J* = 4.0, 3.6 Hz), 4.18 (m, 1H), 3.82 (s, 6H), 3.54 (m, 1H), 3.39 (m, 1H), 2.76 (d, 1H, J = 3.6 Hz), 1.38 (s, 3H), 0.95 (s, 9H), 0.15 (s, 6H). ^13^C NMR (101 MHz, CDCl_3_) *δ*: 163.1, 158.6, 150.3, 144.4, 135.6, 135.2, 135.1, 130.1, 128.1, 127.3, 113.3, 111.4, 87.8, 87.1, 83.9, 75.8, 71.5, 63.1, 55.2, 25.6, 18.2, 11.6, −4.7, −5.1. MS (ESI) calculated for C_37_H_46_N_2_O_8_Si: 674.30. found (M+Na)^+^: 697.74.

**5′-*O*-DMTr-2′-*O*-TBDMS-5-methyl-N^4^-benzoylcytidine (3b).** Compound **2b** was subjected to a similar procedure to that described for preparation of **3a** to give **3b** (1.95 g, 50%). ^1^H NMR (400 MHz, DMSO-*d6*) *δ*: 13.01 (s, 1H), 8.19 (d, 2H), 7.86 (s, 1H), 7.62 (t, 1H, *J* = 7.6, 7.2 Hz), 7.50 (t, 2H, *J* = 7.6, 7.2 Hz), 7.44 (d, 2H, *J* = 7.6 Hz), 7.29–7.38 (m, 7H), 6.95 (d, 4H, *J* = 7.6 Hz), 5.84 (d, 1H, J = 3.6 Hz), 5.25 (d, 1H, *J* = 6.0 Hz), 4.36 (m, 1H), 4.18 (m, 1H), 4.11 (m, 1H), 3.77 (s, 6H), 3.28 (m, 2H), 1.60 (s, 3H), 0.90(s, 9H), 0.12 (s,6H). ^13^C NMR (101 MHz, DMSO-*d6*) *δ*: 158.6, 144.5, 141.7, 135.6, 133.8, 130.1, 128.9, 128.1, 127.8, 127.0, 123.2, 113.2, 88.4, 86.6, 84.3, 75.7, 71.5, 63.0, 55.1, 25.6, 17.9, −4.9, −5.3. MS (ESI) calculated for C_44_H_51_N_5_O_8_Si: 777.34. found (M+H)^+^: 778.21.

**5′-*O*-DMTr-3′-*O*-TBDMS-5-methyl-N^4^-benzoylcytidine (4b).** Compound **2b** was subjected to a similar procedure to that described for preparation of **4a** to give **4b** (1.44g, 37%). ^1^H NMR (400 MHz, DMSO-*d6*) *δ*: 12.98 (s, 1H), 8.17 (d, 1H, *J* = 7.6 Hz), 7.82 (s, 1H), 7.60 (t, 1H, *J* = 7.2 Hz), 7.49 (t, 2H, *J* = 7.6, 7.2 Hz), 7.42 (d, 2H, *J* = 7.6 Hz), 7.28–7.36 (m, 7H), 6.92 (d, 4H, *J* = 7.6 Hz), 5.82 (1H), 5.22 (d, 1H, *J* = 5.6 Hz), 4.34 (1H), 4.09–4.16 (m, 2H), 3.75 (s, 6H), 3.28 (m, 2H), 1.58 (s, 3H), 0.88(s, 9H), 0.09 (s, 6H). ^13^C NMR (101 MHz, DMSO-*d6*) *δ*: 158.4, 144.3, 141.8, 135.2, 132.7, 130.1, 128.7, 113.3, 89.3, 86.8, 85.1, 74.8, 72.0, 62.8, 55.4, 25.8, 18.1, −4.2, −5.3. MS (ESI) calculated for C_44_H_51_N_5_O_8_Si: 777.34. found (M+H)^+^: 778.21.

**2′-*O*-TBDMS-5-metheyluridine (5a).** To a solution of **3a** (1.35 g, 2 mmol) in CH_2_Cl_2_ (20 mL) was added 10-mL DCA solution (3% in CH_2_Cl_2_) to remove the 4,4′-dimethoxytriphenylmethyl group. The mixture was stirred for 5min. and then washed with H_2_O (4 × 20 mL). The organic layer was dried over anhydrous Na_2_SO_4_ and filtered and concentrated under reduced pressure. The residue was purified by SiO_2_ gel chromatography to give **5a** (0.68 g, 92%)**.** ^1^H NMR (400 MHz, DMSO-*d6*) *δ*: 11.31 (s, 1H), 7.82 (s, 1H), 5.78 (d, 1H, *J* = 4.8 Hz), 5.18 (m, 1H), 4.95 (d, 1H, *J* = 5.2 Hz), 4.14 (m, 1H, *J* = 4.4 Hz), 3.96 (d, 1H, *J* = 4.8 Hz), 3.87 (m, 1H), 3.57–3.73 (m, 2H), 1.77 (s, 3H), 0.83 (s, 9H), 0.03 (s, 3H), 0.01 (s, 3H). ^13^C NMR (101 MHz, DMSO-*d6*) *δ*: 164.1, 151.2, 136.5, 109.7, 87.9, 85.2, 75.9, 69.9, 60.9, 55.3, 26.1, 18.4, 12.6, −4.4, −4.7. MS (ESI) calculated for C_16_H_28_N_2_O_6_Si: 372.17. found (M+Na)^+^: 395.44.

**3′-*O*-TBDMS-5-metheyluridine (9a).** Compound **4a** was subjected to a similar procedure to that described for the preparation of **5a** to give **9a** (0.67 g, 91%). ^1^H NMR (400 MHz, DMSO-*d6*) *δ*: 11.31 (s, 1H), 7.70 (s, 1H), 5.78 (d, 1H, *J* = 6.0Hz), 5.20 (d, 1H, J = 5.6Hz), 5.15 (1H), 4.09–4.12 (m, 2H), 3.83 (m,1H), 3.51–3.63 (m, 2H), 1.78 (s, 3H), 0.88 (s, 9H), 0.09 (s, 6H). ^13^C NMR (101 MHz, DMSO-*d6*) *δ*: 164.2, 151.4, 136.8, 110.0, 87.4, 86.1, 73.0, 72.8, 61.4, 26.3, 18.5, 12.7, −4.0, −4.5. MS (ESI)S calculated for C_16_H_28_N_2_O_6_Si: 372.17. found (M+Na)^+^: 395.44.

**2′-*O*-TBDMS-5-methyl-N^4^-benzoylcytidine (5b).** Compound **3b** was subjected to a similar procedure to that described for the preparation of **5a** to give **5b** (0.85 g, 93%). ^1^H NMR (400 MHz, DMSO-*d6*) *δ*: 13.00 (s, 1H), 8.27 (s, 1H), 8.18 (2H), 7.60 (t, 1H, *J* = 7.2, 6.8 Hz), 7.50 (t, 2H, *J* = 7.6, 7.2 Hz), 5.32 (s, 1H), 5.05 (d, 1H, *J* = 5.6 Hz), 4.21 (1H), 4.02 (m, 1H, *J* = 5.2 Hz), 3.93 (d, 1H, *J* = 3.2 Hz), 3.79 (d, 1H), 3.64 (d, 1H), 3.17(d, 1H, *J* = 4.8 Hz), 2.01 (s, 3H), 0.87(s, 9H), 0.07 (s,6H). ^13^C NMR (101 MHz, DMSO-*d6*) *δ*: 132.9, 130.0, 128.7, 84.7, 76.4, 68.9, 60.1, 55.4, 49.1, 26.2, 18.4, 0.6, −4.3, −4.6. MS (ESI) calculated for C_23_H_33_N_3_O_6_Si: 475.21. found (M+Na)^+^: 498.44.

**3′-*O*-TBDMS-5-methyl-N^4^-benzoylcytidine (9b).** Compound **4b** was subjected to a similar procedure to that described for the preparation of **5a** to give **9b** (0.88 g, 96%). ^1^H NMR (400 MHz, DMSO-*d6*) *δ*: 12.89 (s, 1H), 8.17 (d, 2H, *J* = 7.2 Hz), 8.13 (s, 1H), 7.60 (t, 1H, *J* = 7.6, 7.2 Hz), 7.50 (t, 2H, *J* = 7.6, 7.2 Hz), 5.81 (d, 1H, *J* = 4.8 Hz), 5.38 (d, 2H), 3.90 (1H), 3.69 (m, 1H,), 3.55 (m, 1H), 3.16 (d, 2H, *J* = 4.4 Hz), 2.02 (s, 3H), 0.89(s, 9H), 0.10 (s, 6H). ^13^C NMR (101 MHz, DMSO-*d6*) *δ*: 133.0, 129.7, 128.9, 86.3, 73.6, 72.3, 60.8, 48.8, 26.2, 18.4, 13.7, 0.6, −4.0, −4.5. MS (ESI) calculated for C_23_H_33_N_3_O_6_Si: 475.21. found (M+Na)^+^: 498.44.

**(3′-O-TBDMS-2′-O-cyanoethylphosphotriester-N^4^-isobutyrylguanosine)-(2′,5′)-(2′-O-TBDMS-5-methyluridine) (6a)**. To a solution of phosphoramidite reagent G (0.48 g, 0.5 mmol) and 1*H*-tetrazole (39 mg, 0.5 mmol) in dry CH_3_CN (5 mL) was added **5a** (0.19 g, 0.5 mmol). The mixture was stirred for 2 h under argon atmosphere to proceed the coupling reaction. Then tBuOOH solution (5.5 M in decane, 1 mmol) was added to the reaction in order to oxide the phosphite trimester to the phosphodiester bond, and the mixture was stirred for 30 min. Then, 0.5-M NaHSO_3_ aqueous solution 5 mL was added to consume the excess tBuOOH and stirred for 10 min, concentrating the solution to oil under reduced pressure. The residue was treated with 3% DCA in CH_2_Cl_2_ (50 mL) for 15 min. The reaction was quenched with MeOH and pyridine. The solvents were removed in vacuo, and the residue was purified by silica gel column chromatography, using CH_2_Cl_2_/MeOH as the eluent, to give intermediate **6a** as a white foam (0.28 g, 60%). Compound **6a** was highly hygroscopic and, thus, was immediately used in the next step.

**(3′-O-TBDMS-2′-O-cyanoethylphosphorothioate-triester-N^4^-isobutyrylguanosine]-(2′,5′)-(2′-O-TBDMS-5-methyluridine) (6b).** To a solution of phosphoramidite reagent G (0.48 g, 0.5 mmol) and 1*H*-tetrazole (39 mg, 0.5 mmol) in dry CH_3_CN (5 mL) was added **5a** (0.19 g, 0.5 mmol). The mixture was stirred for 2 h under argon atmosphere to proceed the coupling reaction. Then, a solution of phenylacetyl disulfide (PADS) (0.30 g, 1 mmol) was added to the reaction in order to oxide the phosphite trimester to the phosphorothioate bond, and the mixture was stirred for 30 min. Then, the solution was concentrated to oil under reduced pressure. The residue was treated with 3% DCA in CH_2_Cl_2_ (50 mL) for 15 min. The solvents were removed in vacuo, and the residue was purified by silica gel column chromatography, using CH_2_Cl_2_/MeOH as the eluent, to give intermediate **6b** as a white foam (0.32 g, 67%). Compound **6b** was highly hygroscopic and, thus, was immediately used in the next step.

**(3′-O-TBDMS-2′-O-cyanoethylphosphotriester-N^4^-isobutyrylguanosine)-(2′,5′)-(2′-O-TBDMS-5-methyl-N^4^-benzoylcytidine) (6c).** Compound **5b** and phosphoramidite reagent G were subjected to a similar procedure to that described for the preparation of **6a** to give **6c** (0.38 g, 72%).

**(3′-O-TBDMS-2′-O-cyanoethylphosphorothioate-triester-N^4^-isobutyrylguanosine)-(2′,5′)-(2′-O-TBDMS-5-methyl-N^4^benzoylcytidine) (6d)**. Compound **5b** and phosphoramidite reagent G were subjected to a similar procedure to that described for the preparation of **6b** to give **6d** (0.42 g, 78%).

**(2′-O-TBDMS-3′-O-cyanoethylphosphotriester-N^6^-benzoyladenosine)-(3′,5′)-(3′-O-TBDMS-5-methyluridine) (10a).** Compound **9a** and phosphoramidite reagent A were subjected to a similar procedure to that described for the preparation of **6a** to give **10a** (0.30 g, 62%).

**(2′-O-TBDMS-2′-O-cyanoethylphosphorothioate-triester-N^6^-benzoyladenosine)-(3′,5′)-(3′-O-TBDMS-5-methyluridine) (10b).** Compound **9a** and phosphoramidite reagent A were subjected to a similar procedure to that described for the preparation of **6b** to give **10b** (0.32 g, 65%).

**(2′-O-TBDMS-3′-O-cyanoethylphosphotriester-N^6^-benzoyladenosine)-(3′,5′)-(3′-O-TBDMS-5-methyl-N^4^-benzoylcytidine) (10c).** Compound **9b** and phosphoramidite reagent A were subjected to a similar procedure to that described for the preparation of **6a** to give **10c** (0.38 g, 70%).

**(2′-O-TBDMS-2′-O-cyanoethylphosphorothioate-triester-N^6^-benzoyladenosine)-(3′,5′)-(3′-O-TBDMS-5-methyl-N^4^-benzoylcytidine) (10d)**. Compound **9a** and phosphoramidite reagent A were subjected to a similar procedure to that described for the preparation of **6b** to give **10d** (0.33 g, 60%).

**(2′3′)cyclic-(3′-O-TBDMS-3′-O-cyanoethylphosphodiester-N^4^-isobutyrylguanosine)-(3′-O-TBDMS-2′-cyanoethylphosphotriester-5-metheyluridine) (7a)**. To a solution of **6a** (0.19 g, 0.2 mmol) in dry CH_3_CN (5 mL) was added 1*H*-tretazole (23 mg, 0.3 mmol). The mixture was stirred for 10 min, and then, 2-cyanoethyl-N,N,N’,N’-tetraisopropyl phosphorodiamidite (78 mg, 0.26 mmol) was dropped in slowly; the mixture was stirred for 4 h under argon atmosphere at room temperature to promote the cyclization. To the mixture was then added tBuOOH solution (5.5 M in decane, 0.3 mmol) and stirred for further 20 min. The reaction was quenched by the addition of 0.5-M NaHSO_3_ aqueous solution (10 mL) and then extracted with CH_2_Cl_2_ (2 × 15 mL). The combined organic layers were dried over anhydrous Na_2_SO_4_, filtered and concentrated under reduced pressure to afford crude intermediate-contained fully protected cyclic dinucleotides **7a**, which was used in the next step without any further purification.

**(2′3′)cyclic-(3′-O-TBDMS-3′-O-cyanoethylphosphorothioatetriester-N^4^-isobutyrylguanosine)-(3′-O-TBDMS-2′-cyanoethylphosphorothioatetriester-5-metheyluridine) (7b)**. To a solution of **6a** (0.19 g, 0.2 mmol) in dry CH_3_CN (5 mL) was added 1*H*-tretazole (23 mg, 0.3 mmol). The mixture was stirred for 10min, and then, 2-cyanoethyl-N,N,N’,N’-tetraisopropyl phosphorodiamidite (78 mg, 0.26 mmol) was dropped in slowly; the mixture was stirred for 4 h under argon atmosphere at room temperature to promote the cyclization. To the mixture was then added PADS (93 mg, 0.3 mmol) and stirred for further 20 min. Then, the solution was concentrated to oil under reduced pressure. The residue was extracted three times with a 1:1 (*v*/*v*) mixture of CH_2_Cl_2_/H_2_O (45 mL). The combined organic layers were pooled, dried over anhydrous Na_2_SO_4_, filtered and concentrated under reduced pressure to afford crude intermediate-contained fully protected cyclic dinucleotides **7b**, which was used in the next step without any further purification.

**(2′3′)cyclic-(3′-O-TBDMS-3′-O-cyanoethylphosphodiester-N^4^-isobutyrylguanosine)-(3′-O-TBDMS-2′-cyanoethylphosphotriester-5-methey-N^4^-benzoylcytidine) (7c)**. Compound **6c** was subjected to a similar procedure to that described for the preparation of **7a** to give crude intermediate-contained fully protected cyclic dinucleotides **7c**, which was used in the next step without any further purification.

**(2′3′)cyclic-(3′-O-TBDMS-3′-O-cyanoethylphosphorothioatetriester-N4-isobutyrylguanosine)-(3′-O-TBDMS-2′-cyanoethylphosphorothioatetriester-5-methey-N^4^-benzoylcytidine) (7d)**. Compound **6d** was subjected to a similar procedure to that described for the preparation of **7b** to give crude intermediate-contained fully protected cyclic dinucleotides **7d**, which was used in the next step without any further purification.

**(2′3′)cyclic-(3′-O-TBDMS-3′-O-cyanoethylphosphodiester-5-metheyluridine)-(3′-O-TBDMS-2′-cyanoethylphosphotriester-N^6^-benzoyladenosine) (11a)**. Compound **10a** was subjected to a similar procedure to that described for the preparation of **7a** to give crude intermediate-contained fully protected cyclic dinucleotides **11a**, which was used in the next step without any further purification.

**(2′3′)cyclic-(3′-O-TBDMS-3′-O-cyanoethylphosphorothioatetriester-5-metheyluridine)-(3′-O-TBDMS-2′-cyanoethylphosphorothioatetriester-N^6^-benzoyladenosine) (11b)**. Compound **10b** was subjected to a similar procedure to that described for the preparation of **7b** to give crude intermediate-contained fully protected cyclic dinucleotides **11b**, which was used in the next step without any further purification.

**(2′3′)cyclic-(3′-O-TBDMS-3′-O-cyanoethylphosphodiester-5-methey-N^4^-benzoylcytidine)-(3′-O-TBDMS-2′-cyanoethylphosphotriester-N^6^-benzoyladenosine) (11c)**. Compound **10c** was subjected to a similar procedure to that described for the preparation of **7a** to give crude intermediate-contained fully protected cyclic dinucleotides **11c**, which was used in the next step without any further purification.

**(2′3′)cyclic-(3′-O-TBDMS-3′-O-cyanoethylphosphorothioatetriester-5-methey-N^4^-benzoylcytidine)-(3′-O-TBDMS-2′-cyanoethylphosphorothioatetriester-N6-benzoyladenosine) (11d)**. Compound **10d** was subjected to a similar procedure to that described for the preparation of **7b** to give crude intermediate-contained fully protected cyclic dinucleotides **11d**, which was used in the next step without any further purification. 

**2′,3′-cyclic-guanosine-5-methyluridine (8a**). To a solution of the product containing **7a** from the previous step in 2 mL EtOH was added 5-mL 33% MeNH_2_ in EtOH and stirred at room temperature for 8 h, and then, the solvent was removed. Et_3_N (3 mL), 1-mL anhydrous pyridine and 1 mL Et_3_N^•^3HF were added, and the mixture was stirred at 50 °C for 3 h. Then, the solvent was evaporated and recrystallized in acetone. The deprotected cyclic dinucleotides as triethylammonium salts were purified with the preparative HPLC. (Agela OCTOPUS purification system; preparative column using an ASB C18 column (21.2 × 250 mm) (Agela Technologies); A = water with 50-mM TEAA, B = MeCN; gradient: 0–2 min: 98%A/2% B; 2–20 min: 98%A to 75% A/2% B to 25% B; 20–25 min: 75% A to 0% A/25% B to 100% B; 25–35 min: 0% A to 98%/100% B to 2% B) (Yield 15–35%). The fractions containing the desired compound were pooled, and the solution was concentrated and lyophilized to give 8a (20 mg, 15%) as a white solid. ^1^H NMR (400 MHz, D_2_O) *δ*: 7.89 (m, 2H), 5.89 (d, 1H, *J* = 4.8Hz), 5.67 (s, 1H), 5.56 (m, 1H), 4.56 (m, 2H), 4.55–4.56 (m,4H), 4.33–4.43 (m, 4H), 3.43 (m, 1H), 2.98 (m, 4H), 1.34 (s, 3H); ^31^P NMR (162 MHz, D_2_O) *δ*: −1.26, −2.61; HRMS (ESI-TOF^−^) calculated for C_20_H_25_N_7_O_15_P_2_: 665.0884; found (M-H)^−^ 664.0782.

**2′,3′-cyclic-guanosine(PS)-5-methyluridine(PS) (8b).** Compound **7b** was subjected to a similar procedure to that described for the preparation of **8a** to give **8b** (25 mg, 18%) as a white solid. ^1^H NMR (400 MHz, D_2_O) *δ*: 7.90 (s, 1H), 7.88 (s, 1H), 5.89 (d, 1H, *J* = 8.8 Hz), 5.67 (s, 1H), 5.56 (m, 1H), 4.55 (m, 2H), 4.34–4.43 (m,4H), 4.11–4.21 (m, 4H), 3.43 (m, 1H), 2.96–3.02 (m, 4H), 1.34 (s, 3H); ^31^P NMR (162 MHz, D_2_O) *δ*: 54.25, 53.47. HRMS (ESI-TOF^−^) calculated for C_20_H_25_N_7_O_13_P_2_S_2_: 697.0427; found (M-H)]^−^ 696.0322.

**2′,3′-cyclic-guanosine-5-methylcytidine (8c).** Compound **7c** was subjected to a similar procedure to that described for the preparation of **8a** to give **8c** (31 mg, 23%) as a white solid. ^1^H NMR (400 MHz, D_2_O) *δ*: 7.97 (s, 1H), 7.97 (s, 2H), 7.58 (s, 1H), 6.33 (d, 1H, *J* = 8.0 Hz), 5.93 (d, 1H, *J* = 8.4 Hz), 5.88 (d, 1H, *J* = 8.8 Hz), 5.66 (m, 1H), 5.56 (m, 1H), 5.29(m, 1H), 4.55–4.60 (m, 4H), 4.34–4.45 (m, 4H), 4.07–4.20 (m, 2H), 2.97–3.02 (m, 2H), 1.33 (s, 3H); ^31^P NMR (162 MHz, D_2_O) *δ*: −1.52, −2.07; HRMS (ESI^−^TOF^−^) HRMS (ESI-TOF^−^) calculated for C_20_H_26_N_8_O_14_P_2_: 664.1044; found (M-H)^−^: 663.0967.

**2′,3′-cyclic-guanosine(PS)-5-methylcytidine(PS) (8d).** Compound **7d** was subjected to a similar procedure to that described for the preparation of **8a** to give **8d** (41 mg, 30%) as a white solid. ^1^H NMR (400 MHz, D_2_O) *δ*: 7.93 (s, 1H), 7.86 (s, 2H), 7.53 (s, 1H), 6.28 (d, 1H, *J* = 8.0Hz), 5.89 (d, 1H, *J* = 8.0 Hz), 5.83 (m, 1H), 5.62 (m, 1H), 5.51 (m, 1H), 5.25 (m, 1H), 4.55 (d, 1H, *J* = 4.0 Hz), 4.50–4.53 (m, 2H), 4.30–4.41 (m, 4H), 4.02–4.13 (m, 2H), 2.93–2.99 (m, 2H), 1.29 (s, 3H). ^31^P NMR (162 MHz, D_2_O) *δ*: 54.99, 53.26. HRMS (ESI-TOF^−^) Exact mass for C_20_H_26_N_8_O_12_P_2_S_2_ (M-H)^−^, Calculated 696.0587; found 695.0482.

**2′,3′-cyclic-5-methyluridine-adenosine (12a).** Compound **11a** was subjected to a similar procedure to that described for the preparation of **8a** to give **12a** (20 mg, 16%) as a white solid. ^1^H NMR (400 MHz, D_2_O) *δ*: 8.16(s, 1H), 7.96(s, 1H), 7.79 (s, 1H), 6.18(s, 1H), 5.97 (d, 1H, *J* = 8.4 Hz), 5.49 (m, 1H), 5.06–5.13 (m, 3H), 4.52 (d, 1H, *J* = 4.0Hz), 4.40–4.47 (m, 2H), 4.00–4.21 (m, 4H), 1.37 (s, 3H). ^31^P NMR (162 MHz, D_2_O) *δ*: −2.81, −3.51. HRMS (ESI-TOF^−^) Exact mass for C_20_H_25_N_7_O_14_P_2_ (M-H)^−^, Calculated 649.0935; found 648.0847.

**2′,3′-cyclic-5-methyluridine(PS)-adenosine(PS) (12b).** Compound **11b** was subjected to a similar procedure to that described for the preparation of **8a** to give **12b** (46 mg, 35%) as a white solid. ^1^H NMR (400 MHz, D_2_O) *δ*: 8.14 (s,1H), 7.99 (s, 1H), 7.81 (s, 1H), 6.18(s, 1H), 5.96 (d, 1H, *J* = 8.4 Hz), 5.48 (m, 1H), 5.06–5.13 (m, 4H), 4.52 (d, 1H), 4.40–4.46 (m, 2H), 4.00–4.19 (m, 4H), 1.37 (s, 3H). ^31^P NMR (162 MHz, D_2_O) *δ*: 55.56, 54.56. Exact mass for C_20_H_25_N_7_O_12_P_2_S_2_ (M-H)^−^, Calculated 681.0478; found 680.0377.

**2′,3′-cyclic-5-methylcytidine-adenosine (12c).** Compound **11c** was subjected to a similar procedure to that described for the preparation of **8a** to give **12c** (27 mg, 21%) as a white solid. ^1^H NMR (400 MHz, D_2_O) *δ*: 7.93 (s, 1H), 7.84 (s, 1H), 5.86–5.91 (m, 2H), 5.46 (m, 1H), 4.56–4.59 (m, 2H), 4.40–4.48 (m, 3H), 4.09–4.21 (m, 3H), 1.42 (s, 3H). ^31^P NMR (162 MHz, D_2_O) *δ*: −1.87, −2.93. Exact mass for C_20_H_26_N_8_O_13_P_2_ (M-H)^−^, Calculated 648.1095; found 648.1008.

**2′,3′-cyclic-5-methylcytidine(PS)-adenosine(PS) (12d).** Compound **11d** was subjected to a similar procedure to that described for the preparation of **8a** to give **12d** (30 mg, 22%) as a white solid. ^1^H NMR (400 MHz, D_2_O) *δ*: 7.94 (s, 1H), 7.86 (s, 1H), 5.86–5.91 (m, 2H), 5.46 (m, 1H), 4.57–4.59 (m, 2H), 4.38–4.45 (m, 3H), 4.09–4.21 (m, 3H), 1.46 (s, 3H). ^31^P NMR (162 MHz, D_2_O) *δ*: 53.61, 52.19. Exact mass for C_20_H_26_N_8_O_11_P_2_S(M-H)^−^, Calculated 680.0638; found 679.0547.

### 4.2. SPR

SPR system was primed using the manufacturer’s HEPES-buffered saline at 25 °C. CM5 chips were warmed to room temperature on the bench before being added to the system. SPR surfaces were cleaned and conditioned (separate injections of 50 mM NaOH, 1 M NaCl, 0.1% SDS and 10 mM HCl, each for 60 s at 10 μL/min) prior to normalization using the manufacturer’s 70% glycerol solution (GE Healthcare). STING was prepared at 100 μg/mL in SPR running buffer with no DMSO and injected twice onto channel 2 for 600 s at 5 μL/min. Final density ca. 12,000 RU.

Cyclic dinucleotides were prepared to 1-mM stocks in SPR running buffer. A 12-concentrations dilution series, including a blank containing no substrate, were made and diluted in a 96-well polypropylene Greiner plate. Plates were spun and then placed at 4 °C until experimentation. Experiments were performed at 60 μL/min with data collected every 0.1s.

### 4.3. Stability Assay in Serum and NP1 Solutions

cGAMP and synthesized CDNs (10 μg/mL) were incubated in a solution (60 μL) of an enzyme (2.5-mU NP1 in 3 mM acetate buffer containing 2-mM ZnCl_2_ (pH 5.3)) or serum (10-mM phosphate-buffered saline buffer containing 20% FBS and 1 mM Mg^2+^) in a water bath at 37 °C. At various times, aliquots of the reaction mixture were collected, and degradation was stopped by adding 5 μL EDTA (0.5 M, pH 8.0) and diluted with 60 μL water. Twenty microliters of each aliquot was injected directly into HPLC (Waters 2695 Separations Module equipped with PDA W2998 detector; column Waters SymmetryShield^TM^ RP18 5 μm (4.6 mm × 250 mm), flow rate 1ml/min, detection at 254 nm and autosampler temperature 25 °C) for analysis. TEAA buffer (5 mM) (solvent A) and MeCN (solvent B) were used as the mobile phase. The gradient was set as following: 0–2 min: 98% A; 2–10 min: 98% A to 90% A/2% B to 10% B; 10–15 min: 90% A to 0% A/10% B to 100% B; 15–20 min: 0%A to 0%A/100%B to 100% B;20–25min:0%A to 98%A/100%B to 2%B.

### 4.4. Enzyme-Linked Immunosorbent Assay (ELISA)

The IFN-β levels in the cell culture supernatants were measured using the ELISA kits (Meimian Biotechnology, Jiangsu, China), according to the manufacturer’s instructions.

### 4.5. Total RNAs Isolation, Reverse Transcription and Real-Time Quantitative PCR

Total RNAs were isolated from cells using TRIzol Reagent (Life Technologies, Shanghai, China). RNA concentrations were determined by Nanodrop 1000 (Thermo Scientific, Massachusetts. America). First-strand complementary DNA (cDNA) was synthesized using PrimeScript™ RT Master Mix (Takara Bio, Kusatsu-shi, Japan), according to the manufacturer’s instructions. RT-qPCR was performed in the ABI QuantStudio™ 7Flex Real-Time PCR Detection System with the SYBR™ Select Master Mix (Thermo Fisher, Massachusetts. America), and human β-Actin was used as an internal control. Comparative quantification was determined by using the 2^ΔΔCt^ method. Primers for RT-qPCR are listed as follow:

β-actin forward primer 5′- CATGTACGTTGCTATCCAGGC-3′,

β-actin reverse primer 5′-CTCCTTAATGTCACGCACGAT-3′;

IFN-β forward primer 5′- CCTGAAGGCCAAGGAGTACA-3′,

IFN-β reverse primer 5′- AGCAATTGTCCAGTCCCAGA-3′;

IL6 forward primer 5′- ACTCACCTCTTCAGAACGAATTG-3′,

IL6 reverse primer 5′- CCATCTTTGGAAGGTTCAGGTTG-3′;

CXCL10 forward primer 5′- AGTGGCATTCAAGGAGTACCT-3′,

CXCL10 reverse primer 5′- TGATGGCCTTCGATTCTGGA-3′;

### 4.6. Docking Calculations of **8c** and **8d**

The 2D structure of a small molecule was converted to 3D structures in MOE v2015.1001 through energy minimization. The X-ray structure of protein hSTING was downloaded from RCSB Protein Data Bank (PDB ID: 4LOH). Prior to docking, the force field of AMBER10:EHT and the implicit solvation model of the reaction field (R-field) were selected. The protonation state of the protein and the orientation of the hydrogens were optimized by LigX, at a pH of 7 and temperature of 300 K. MOE-Dock was used for molecular docking. The binding site of the protein was defined around the native ligand in the X-ray structure. The docking workflow followed the “induced fit” protocol, in which the side chains of the receptor pocket were allowed to move according to ligand conformations, with a constraint on their positions. The weight used for tethering side-chain atoms to their original positions was 10. All docked poses were ranked by London dG scoring first; then, a force field refinement was carried out on the top 10 poses, followed by a rescoring of GBVI/WSA dG. The conformation with the lowest free energy of binding was selected as the best (probable) binding mode. Molecular graphics were generated by PyMOL.

## Figures and Tables

**Table 1:** Binding affinities of the cyclic GMP-AMP (cGAMP) analogs with human stimulator of interferon genes (STING).

Compounds	K_a_(1/Ms)	K_d_(1/s)	K_D_(μM)
**8a**	349.4	2.649 × 10^−3^	7.581
**8b**	233.3	1.339 × 10^−4^	0.599
**8c**	152.3	1.397 × 10^−5^	0.092
**8d**	228.3	8.647 × 10^−6^	0.038
**12a**	207.7	2.431 × 10^−3^	11.704
**12b**	289.1	9.865 × 10^−4^	3.412
**12c**	198.2	7.331 × 10^−4^	3.699
**12d**	246.8	2.059 × 10^−4^	0.728
**cGAMP**	211.3	1.147 × 10^−4^	0.543

**Table 2:** The docking scores of OPFV with human STING.

Ligand	Receptor	Docking Score (kcal/mol)
**cGAMP**	hSTING	−9.76
**8c**	hSTING	−10.88
**8d**	hSTING	−11.83

## Supplementary Materials

The following are available online. Figures S1–S18: ^1^H, ^13^C and ^31^P-NMR spectra for the compounds listed in this study.
