# Supplementary material for: Design, Synthesis and Biological Evaluation of (2′,5′ and 3′5′-Linked) cGAMP Analogs that Activate Stimulator of Interferon Genes (STING)
Source: Molecules. 2020 Nov 12;25(22):5285. doi: 10.3390/molecules25225285 (PMC7697705; doi:10.3390/molecules25225285)
Supplement: Supplementary file 1 [file molecules-25-05285-s001.pdf]

Supporting information

Design, Synthesis and Biological Evaluation of (2′,5′ and 3′5′-linked) cGAMP Analogs that Activate Stimulator of Interferon Genes (STING)

Xin Xie[1], Junyi Liu[1,2,*], Xiaowei Wang[1,*]

1 Department of Chemical Biology, School of Pharmaceutical Sciences, Peking University, Beijing 100191, China

2 State Key Laboratory of Natural and Biomimetic Drugs, Peking University, Beijing 100191, China

* Correspondence: xiaoweiwang@bjmu.edu.cn; jyliu@bjmu.edu.cn

¹H-NMR, ¹³C-NMR and ³¹P-NMR spectra

2a:

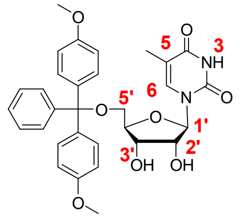

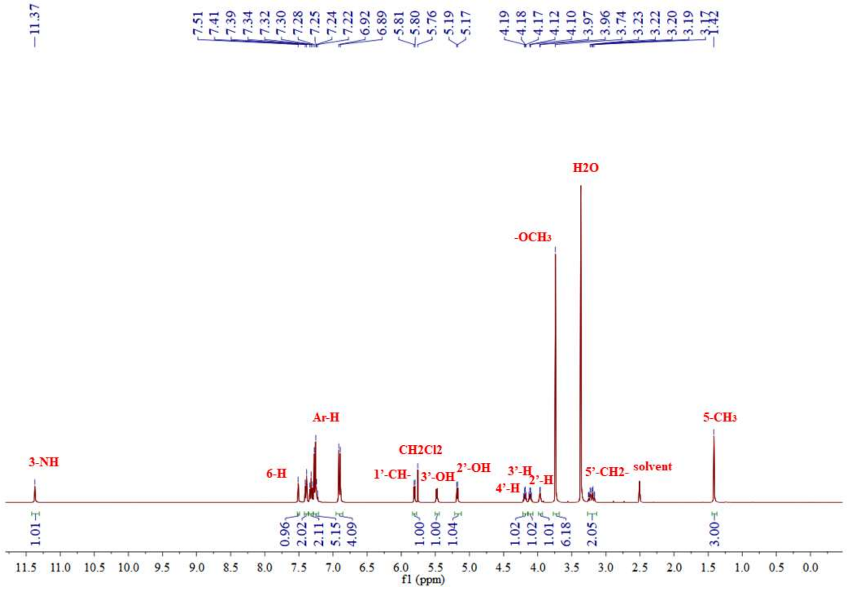

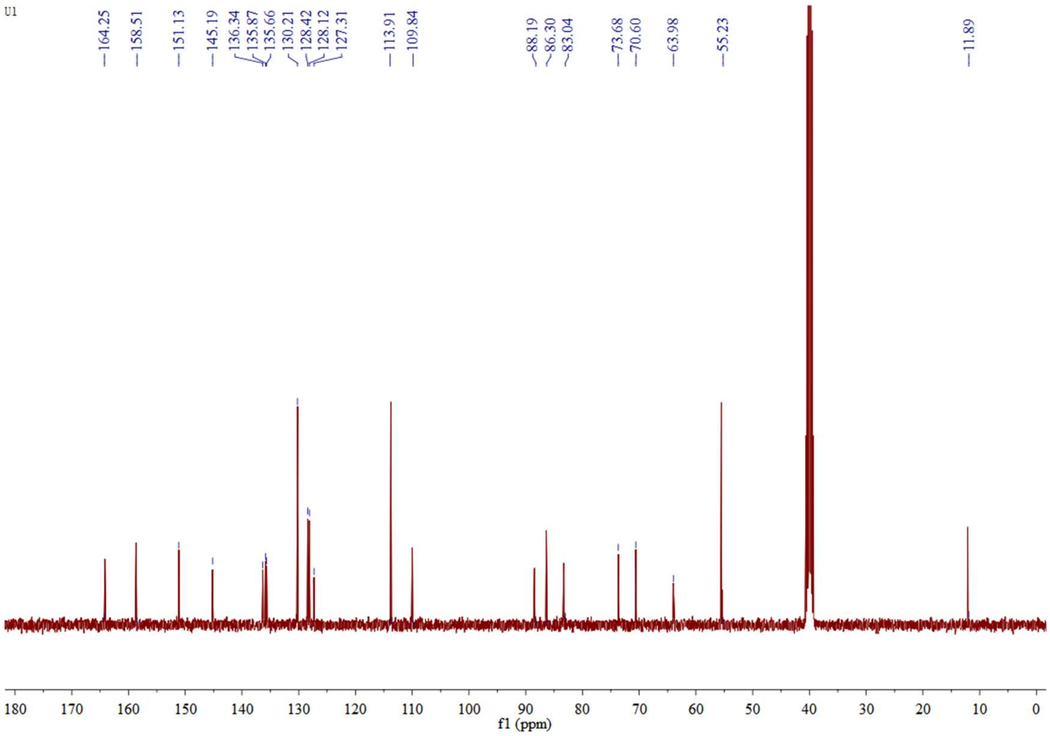

2b:

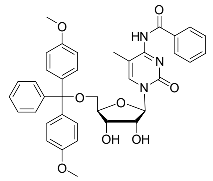

O
HN
N
O
O
O
O
OH
OH
N
O

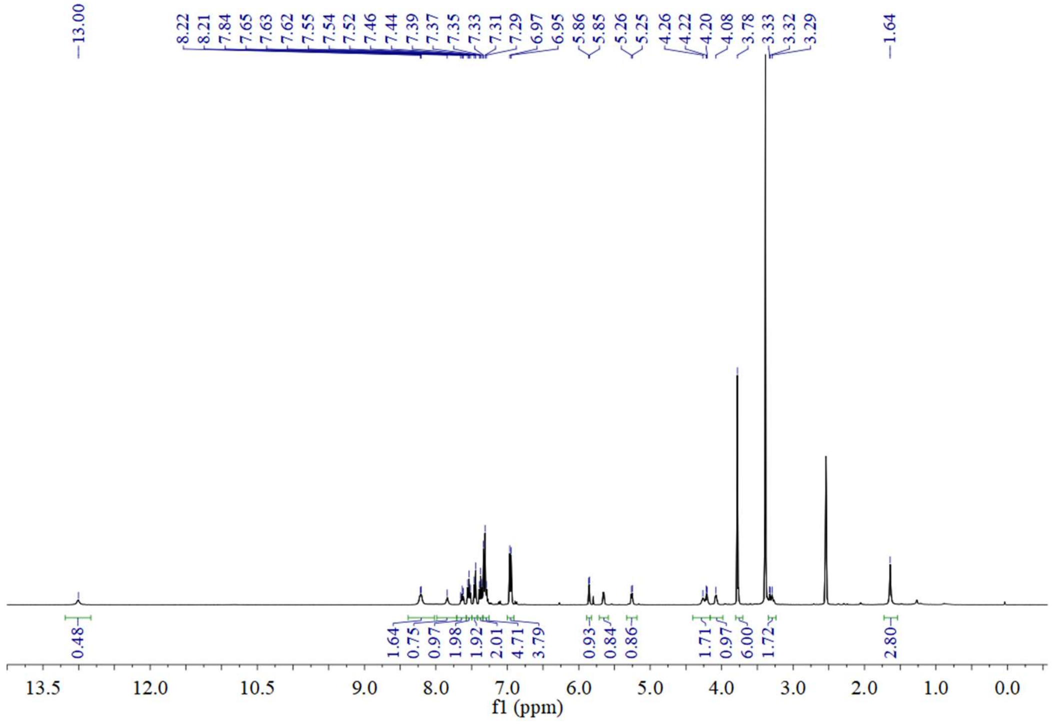

13.00
8.22 8.21 7.84 7.65 7.63 7.62 7.55 7.54 7.52 7.46 7.44 7.39 7.37 7.35 7.33 7.31 7.29 6.97 6.95
5.86 5.85 5.26 5.25 4.26 4.22 4.20 4.08 3.78 3.33 3.32 3.29
1.64
0.48
1.64 0.75 0.97 1.98 1.92 2.01 4.71 3.79
0.93 0.84 0.86
1.71 0.97 6.00 1.72
2.80
13.5 12.0 10.5 9.0 8.0 7.0 6.0 5.0 4.0 3.0 2.0 1.0 0.0
f1 (ppm)

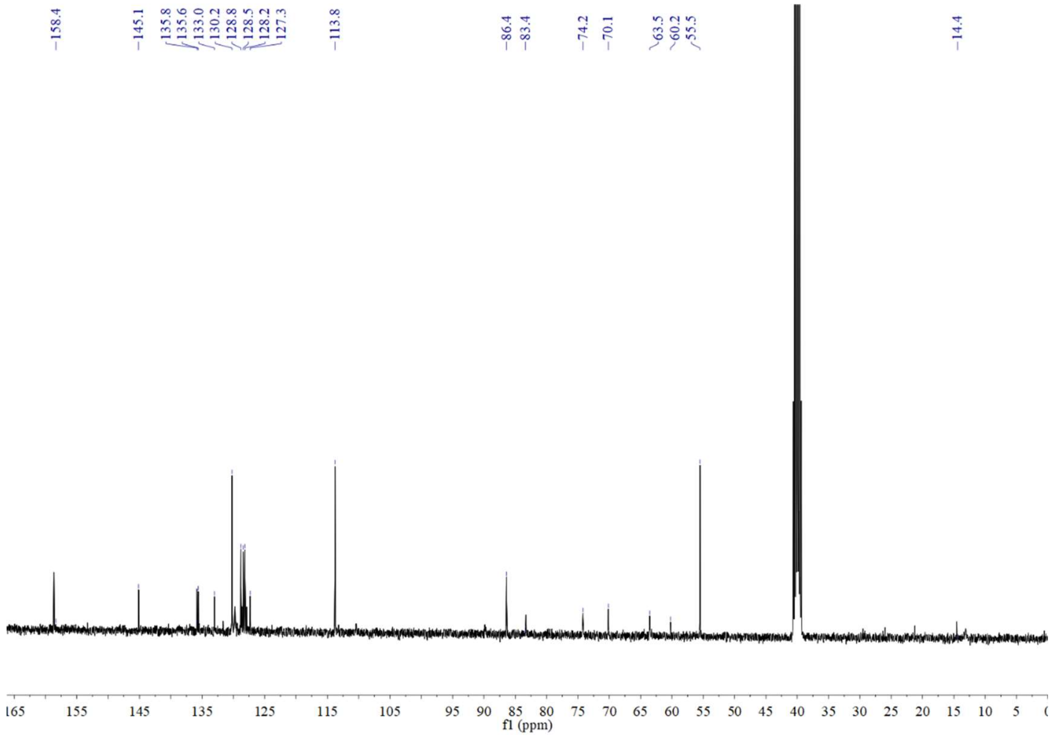

158.4
145.1
135.8 135.6 133.0 130.2 128.8 128.5 128.2 127.3
113.8
86.4 83.4
74.2 70.1
63.5 60.2 55.5
14.4
165 155 145 135 125 115 105 95 90 85 80 75 70 65 60 55 50 45 40 35 30 25 20 15 10 5
f1 (ppm)

3a:

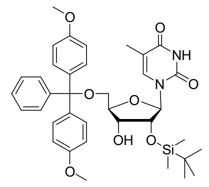

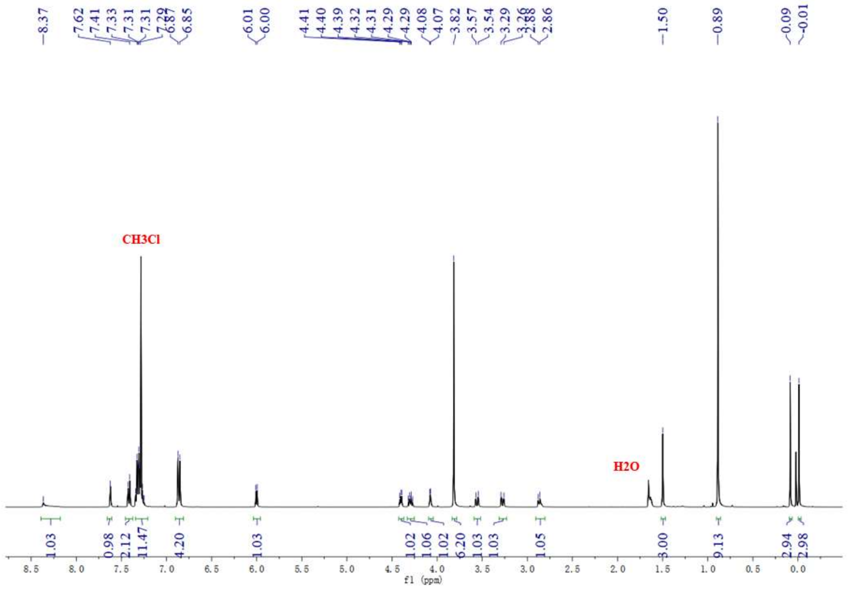

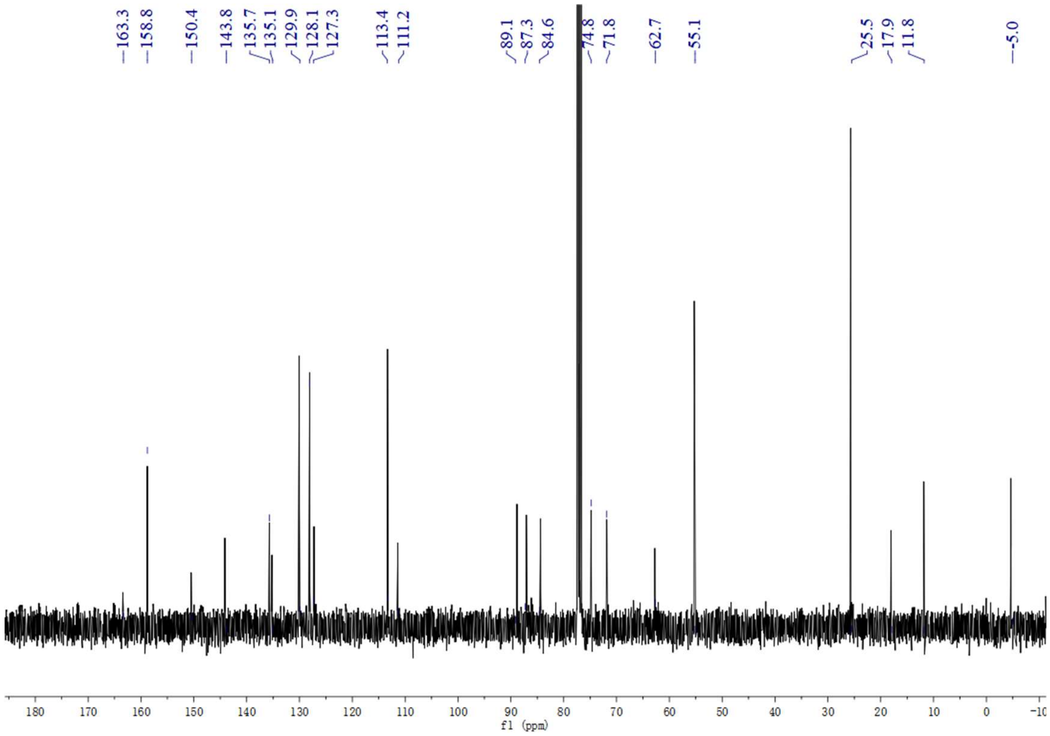

4a:

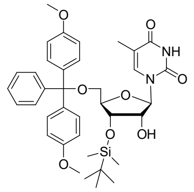
O
NH
O
N
O
O
O
OH
O
Si
O

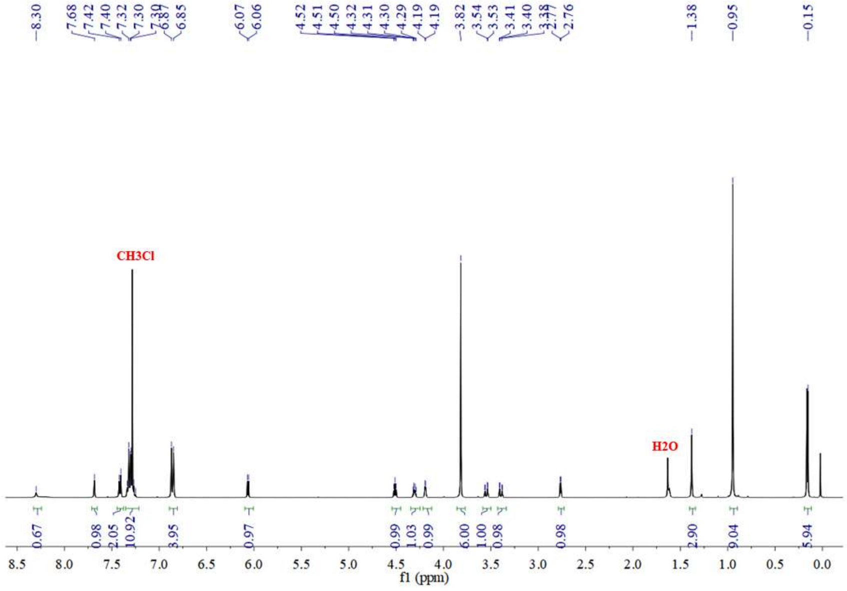
8.30
7.68
7.42
7.40
7.32
7.30
6.87
6.85
6.07
6.06
4.52
4.51
4.50
4.32
4.31
4.30
4.29
4.19
4.19
3.82
3.54
3.53
3.41
3.40
2.76
1.38
0.95
0.15
CH3Cl
H2O
0.67
0.98
2.05
10.92
3.95
0.97
0.99
1.03
0.99
6.00
1.00
0.98
0.98
2.90
9.04
5.94
f1 (ppm)

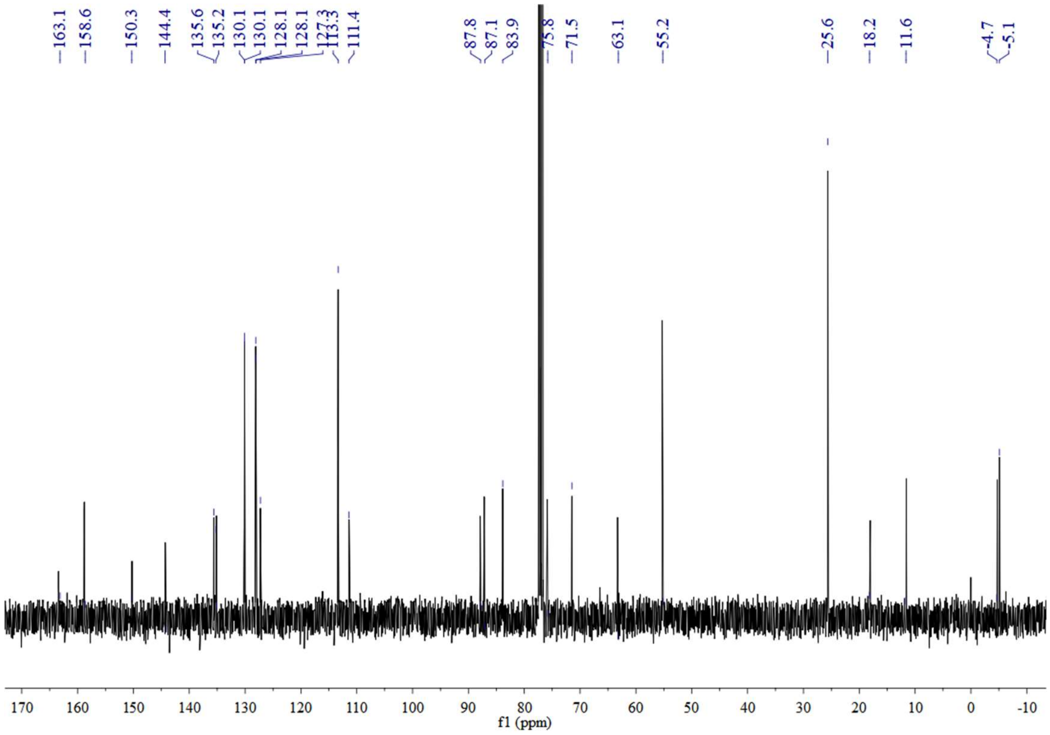
163.1
158.6
150.3
144.4
135.6
135.2
130.1
130.1
128.1
128.1
113.3
111.4
87.8
87.1
83.9
75.8
71.5
63.1
55.2
25.6
18.2
11.6
-4.7
-5.1
f1 (ppm)

3b:

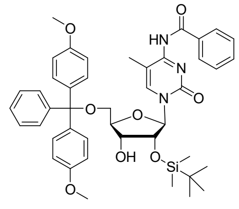
O
HN
N
O
N
O
O
O
OH
O
Si
O

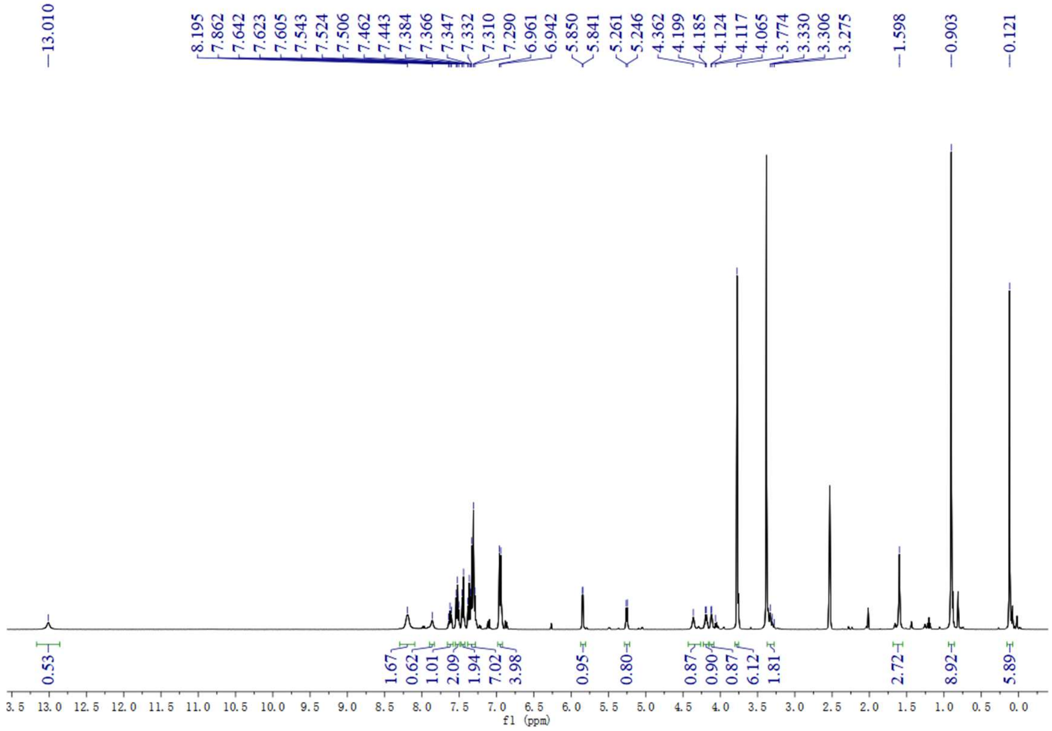
13.010
8.195
7.862
7.642
7.623
7.605
7.543
7.524
7.506
7.462
7.443
7.384
7.366
7.347
7.332
7.310
7.290
6.961
6.942
5.850
5.841
5.261
5.246
4.362
4.199
4.185
4.124
4.117
4.065
3.774
3.330
3.306
3.275
1.598
0.903
0.121
0.53
1.67
0.62
1.01
2.09
1.94
7.02
3.98
0.95
0.80
0.87
0.90
0.87
6.12
1.81
2.72
8.92
5.89
f1 (ppm)

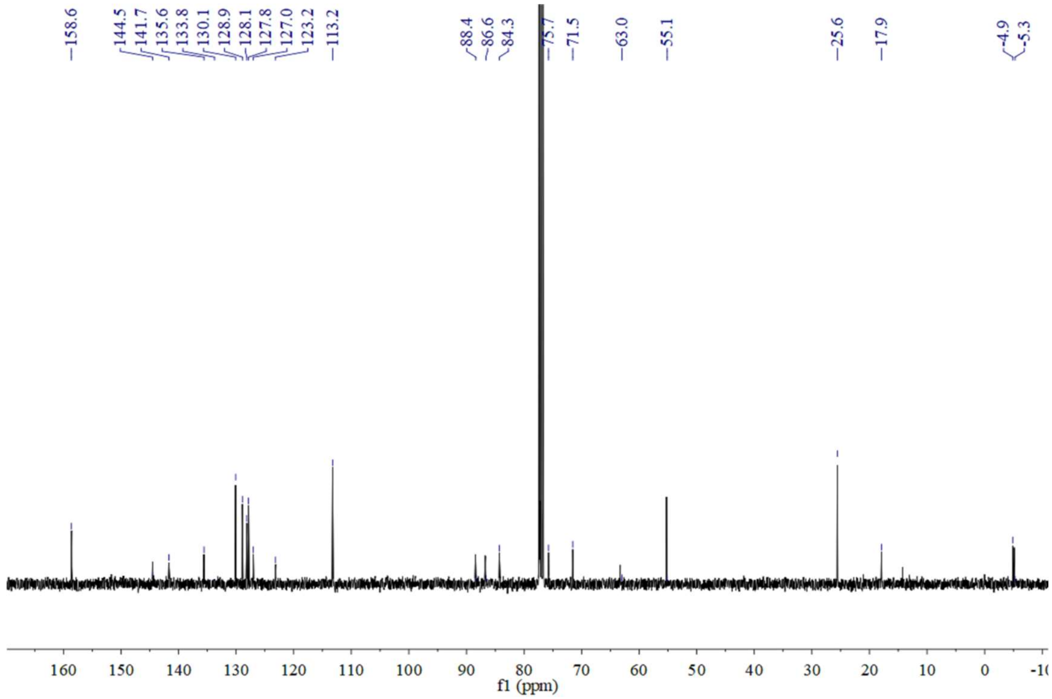
158.6
144.5
141.7
135.6
133.8
130.1
128.9
128.1
127.8
127.0
123.2
113.2
88.4
86.6
84.3
75.7
71.5
63.0
55.1
25.6
17.9
-4.9
-5.3
f1 (ppm)

4b:

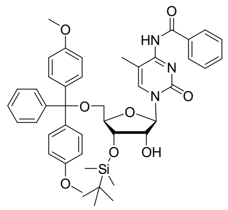

HN
O
N
N
O
O
O
O
O
Si
OH

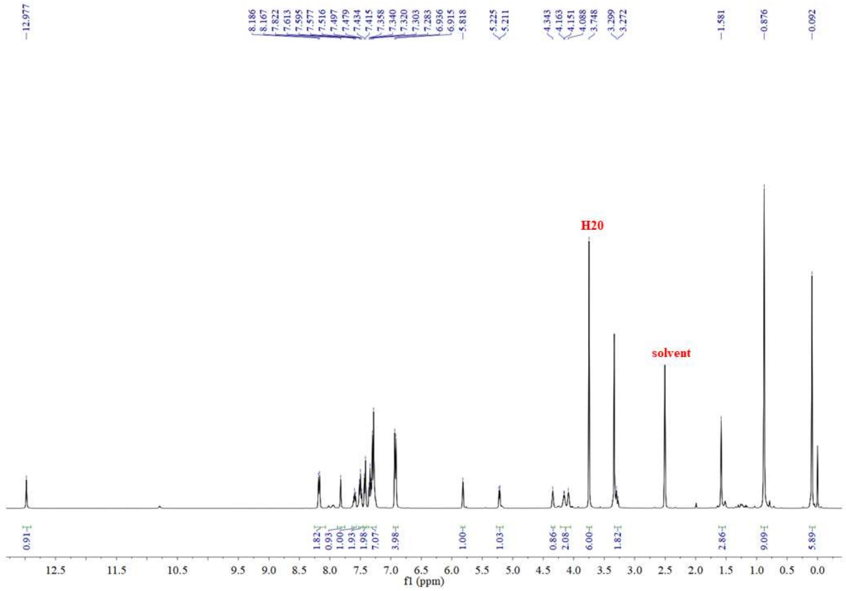

H20
solvent
f1 (ppm)

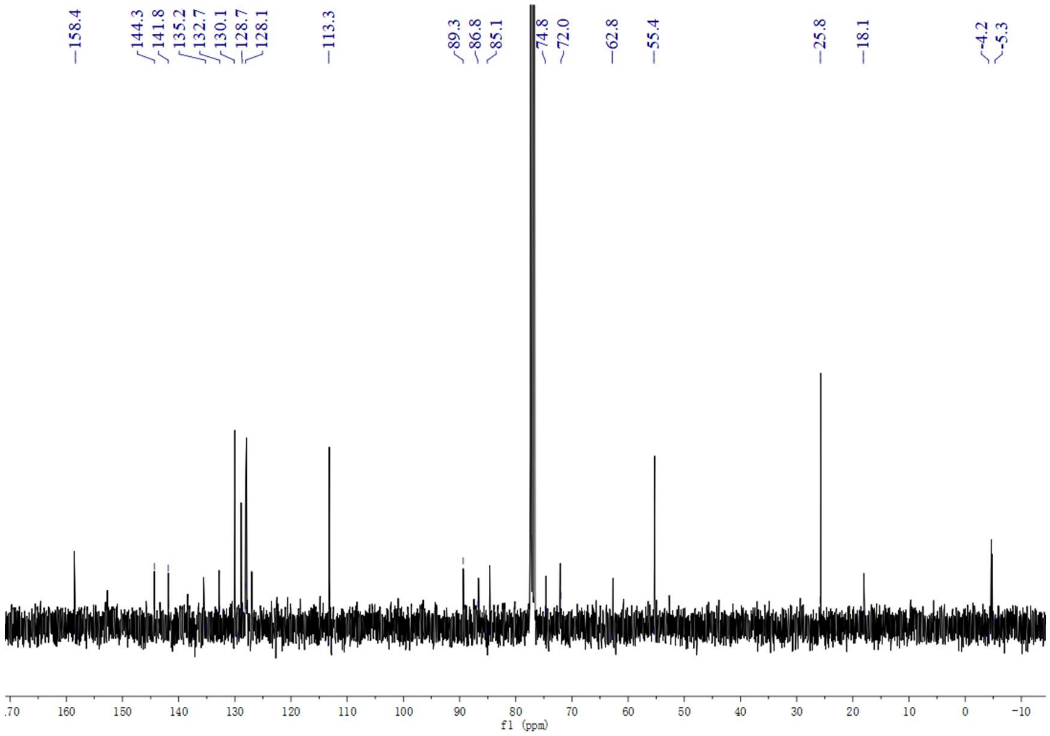

158.4
144.3
141.8
135.2
132.7
130.1
128.7
128.1
113.3
89.3
86.8
85.1
74.8
72.0
62.8
55.4
25.8
18.1
-4.2
-5.3
f1 (ppm)

5a:

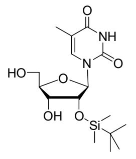

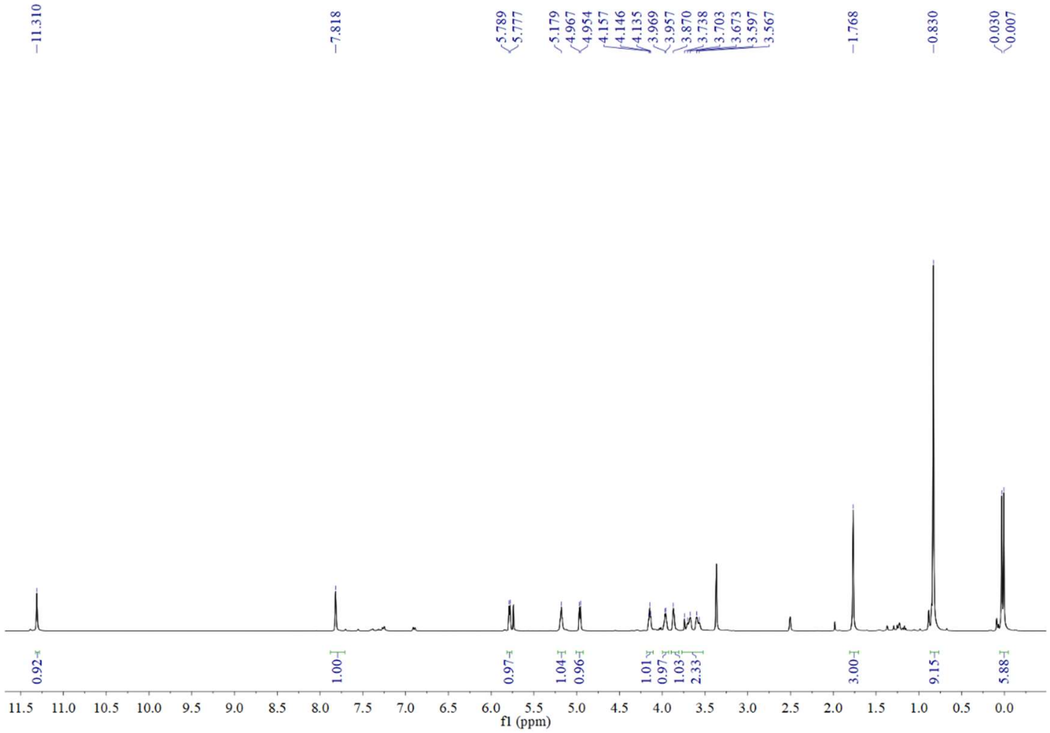

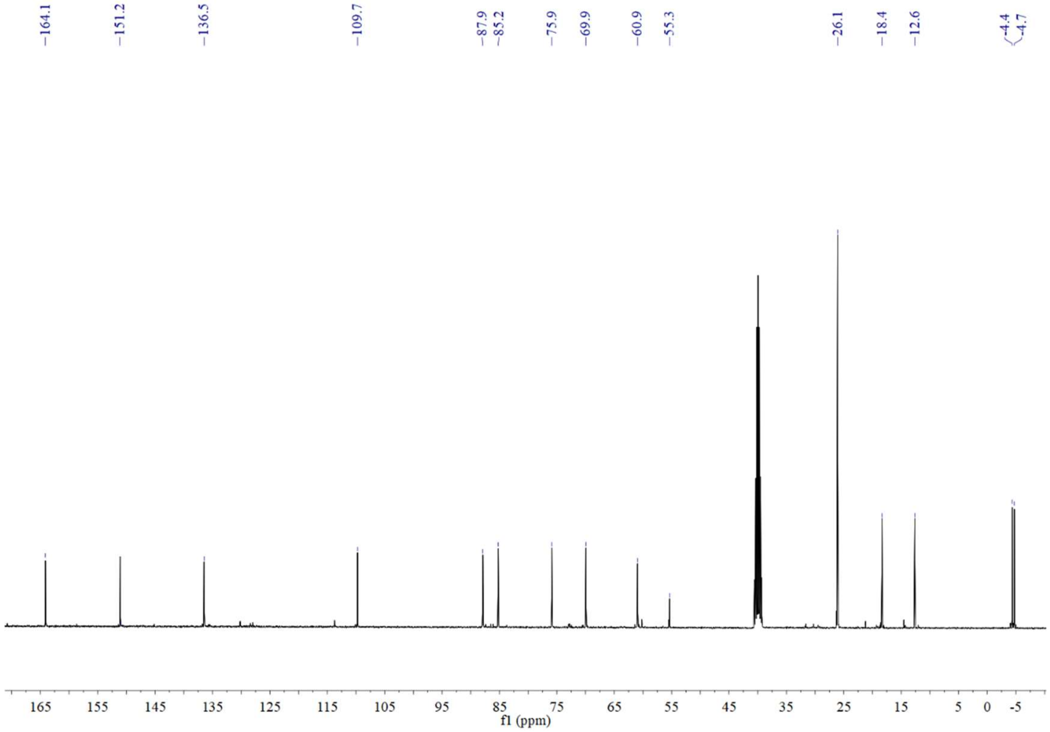

9a:

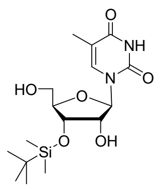
O
NH
O
N
HO
O
O
Si
OH

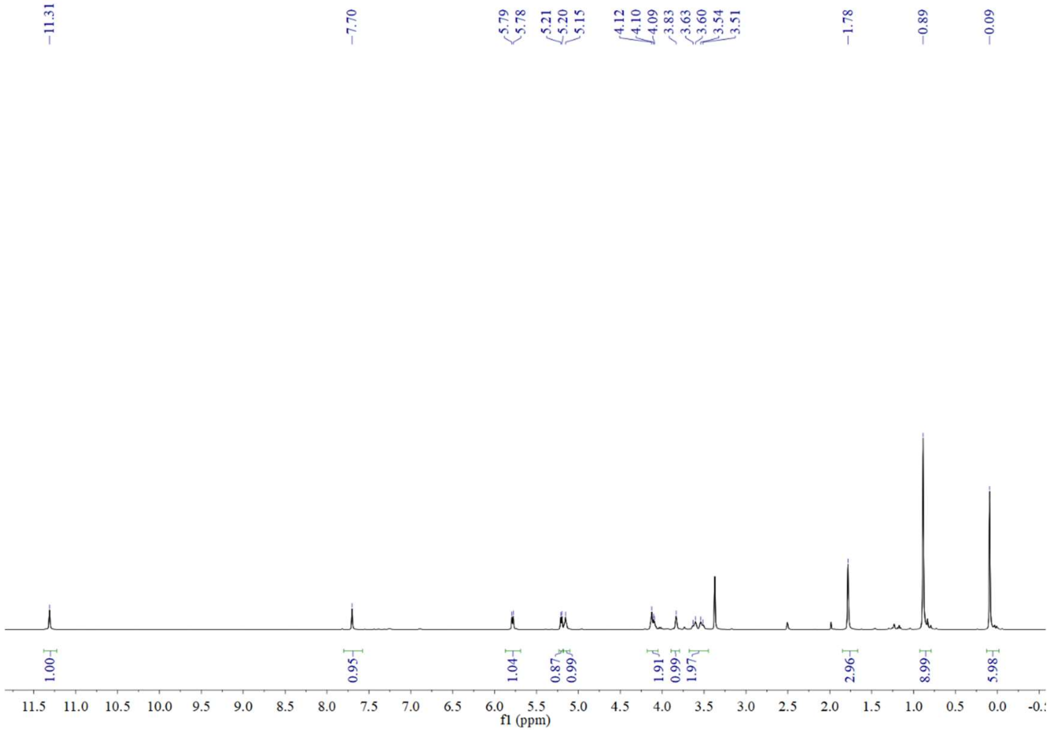
11.31
7.70
5.79
5.78
5.21
5.20
5.15
4.12
4.10
4.09
3.83
3.63
3.60
3.54
3.51
1.78
0.89
0.09
1.00
0.95
1.04
0.87
0.99
1.91
0.99
1.97
2.96
8.99
5.98
11.5 11.0 10.5 10.0 9.5 9.0 8.5 8.0 7.5 7.0 6.5 6.0 5.5 5.0 4.5 4.0 3.5 3.0 2.5 2.0 1.5 1.0 0.5 0.0
f1 (ppm)

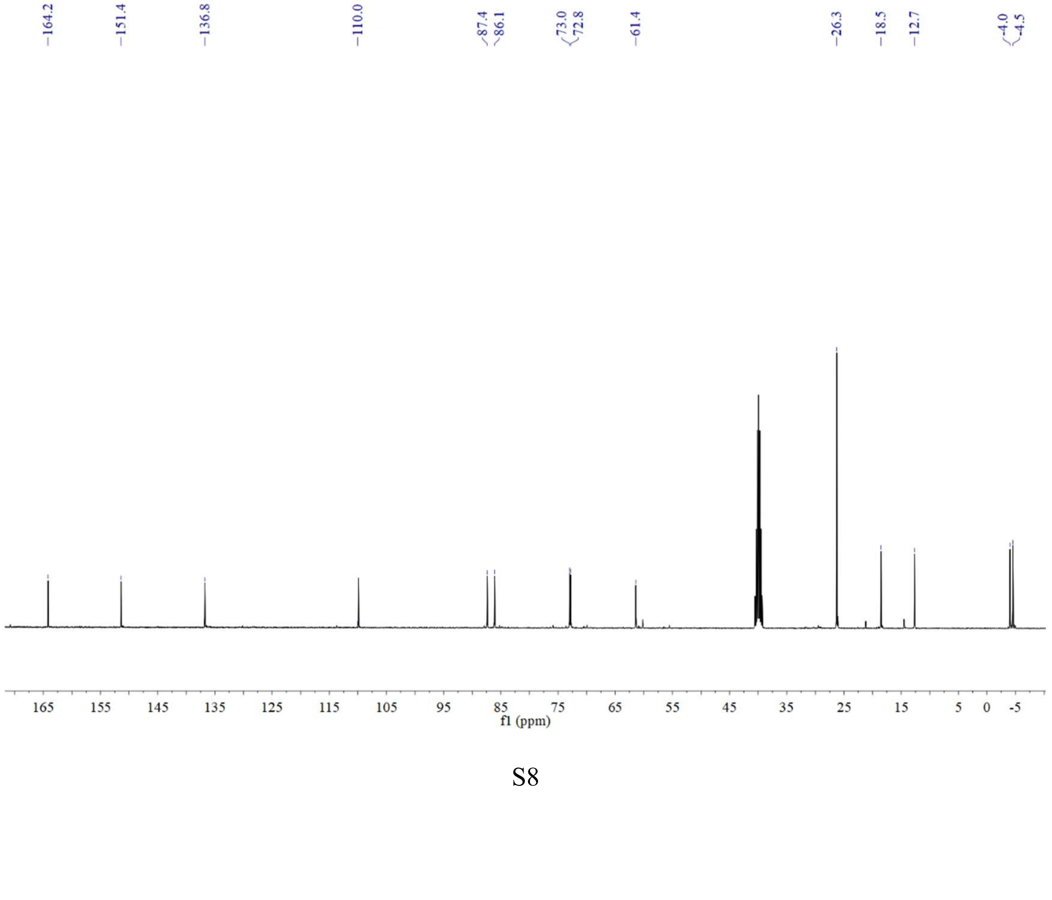
164.2
151.4
136.8
110.0
87.4
86.1
73.0
72.8
61.4
26.3
18.5
12.7
-4.0
-4.5
165 155 145 135 125 115 105 95 85 75 65 55 45 35 25 15 5 0 -5
f1 (ppm)

5b:

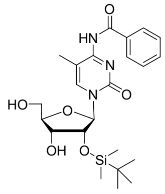

O
HN
N
N
O
HO
O
OH
O
Si

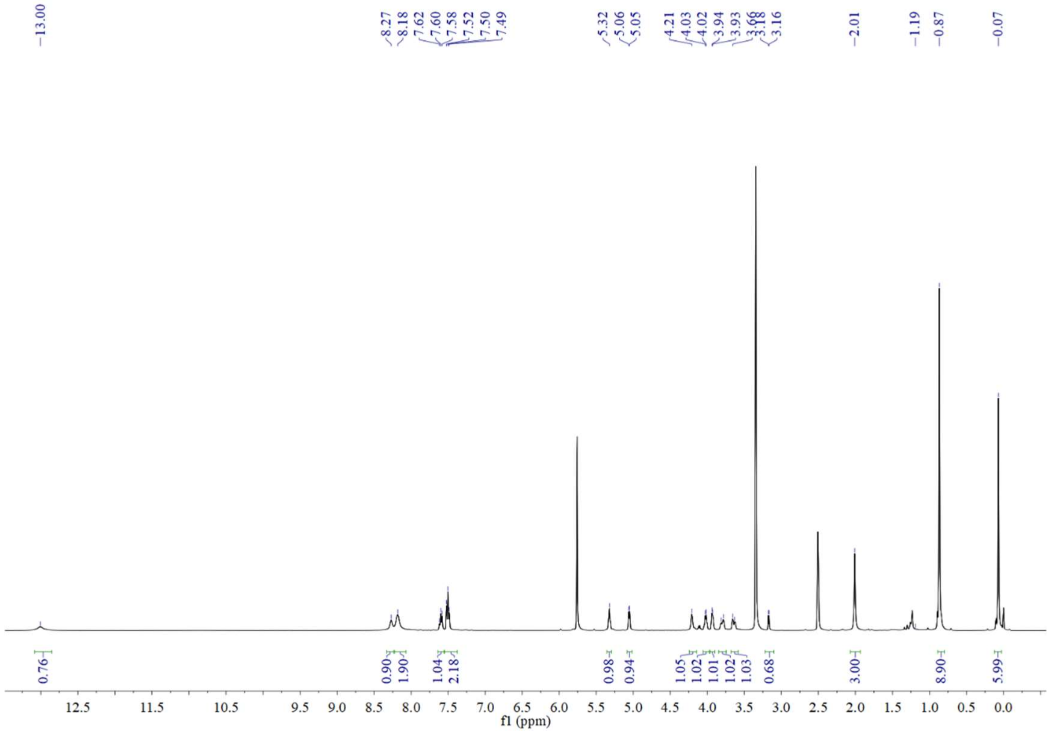

13.00
8.27
8.18
7.62
7.60
7.58
7.52
7.50
7.49
5.32
5.06
5.05
4.21
4.03
4.02
3.94
3.93
3.68
3.18
3.16
2.01
1.19
0.87
0.07
0.76
0.90
1.90
1.04
2.18
0.98
0.94
1.05
1.02
1.01
1.02
1.03
0.68
3.00
8.90
5.99
f1 (ppm)

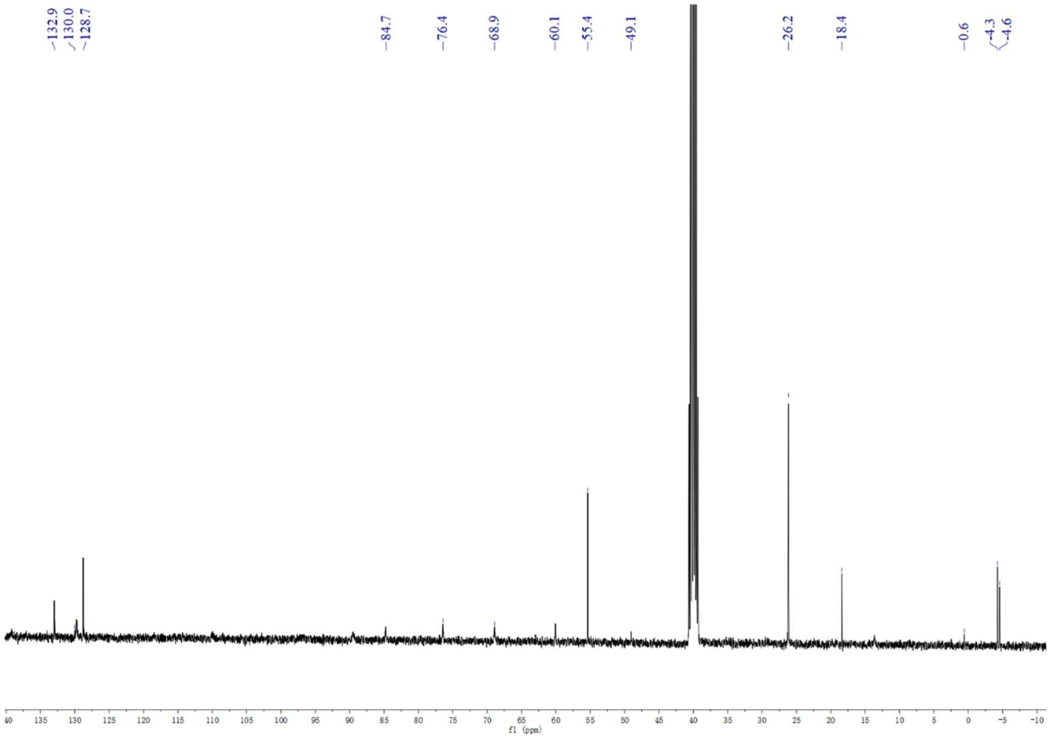

132.9
130.0
128.7
84.7
76.4
68.9
60.1
55.4
49.1
26.2
18.4
0.6
-4.3
-4.6
f1 (ppm)

9b:

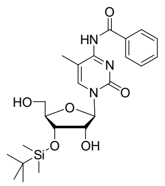
HN
O
N
O
HO
O
N
Si
O
OH

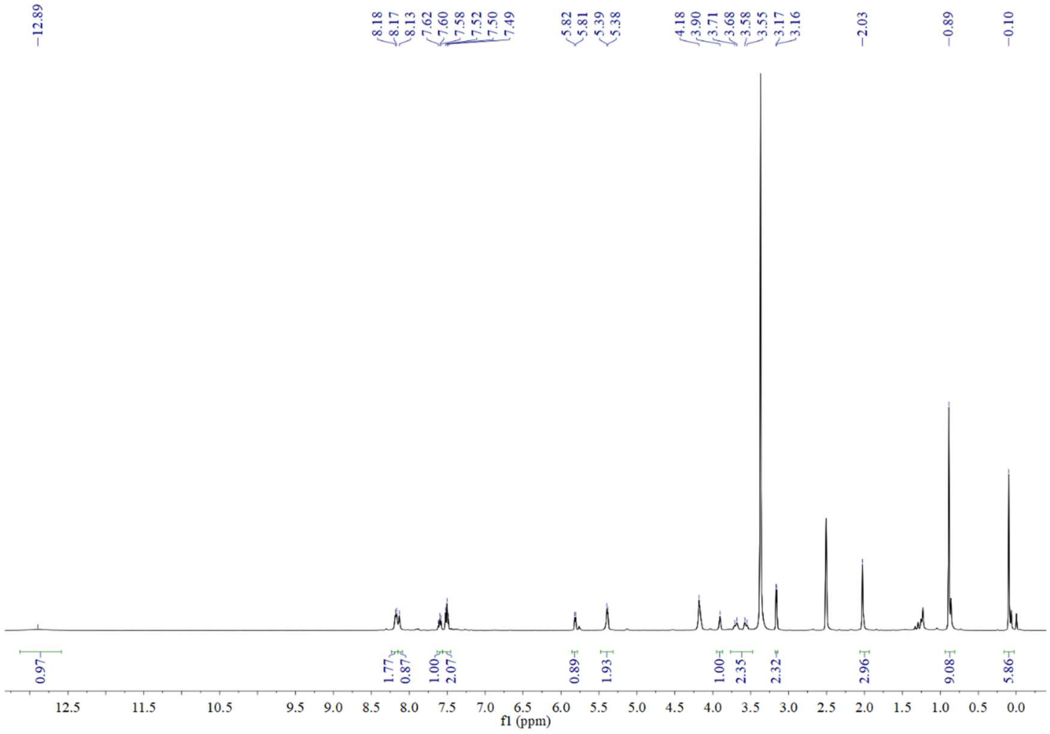
12.89
8.18 8.17 8.13 7.62 7.60 7.58 7.52 7.50 7.49
5.82 5.81 5.39 5.38
4.18 3.90 3.71 3.68 3.58 3.55 3.17 3.16
2.03
0.89
0.10
0.97 1.77 0.87 1.00 2.07 0.89 1.93 1.00 2.35 2.32 2.96 9.08 5.86
f1 (ppm)

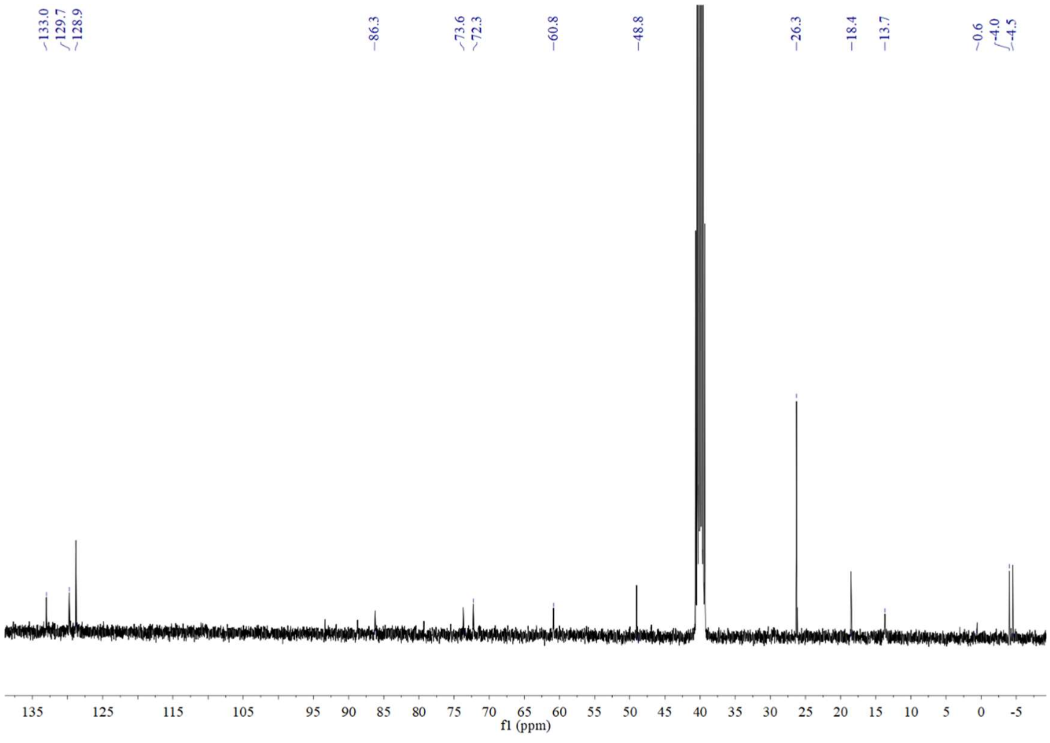
133.0 129.7 128.9
86.3
73.6 72.3
60.8
48.8
26.3
18.4
13.7
0.6 -4.0 -4.5
f1 (ppm)

8a:

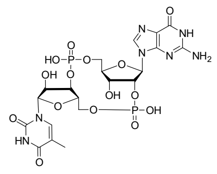
HO
P
O
O
OH
O
O
N
O
HN
O
OH
O
N
N
NH
O
N
NH2
O
P
OH
O

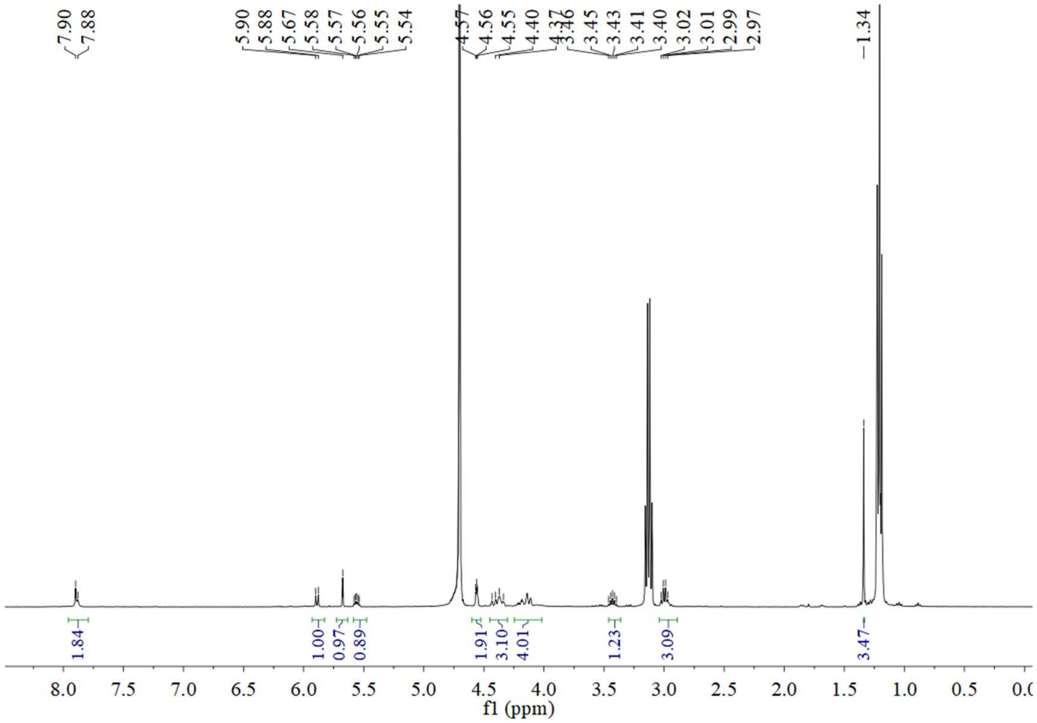
7.90
7.88
5.90
5.88
5.67
5.58
5.57
5.56
5.55
5.54
4.57
4.56
4.55
4.40
4.37
3.46
3.45
3.43
3.41
3.40
3.02
3.01
2.99
2.97
1.34
1.84
1.00
0.97
0.89
1.91
3.10
4.01
1.23
3.09
3.47
8.0
7.5
7.0
6.5
6.0
5.5
5.0
4.5
4.0
3.5
3.0
2.5
2.0
1.5
1.0
0.5
f1 (ppm)

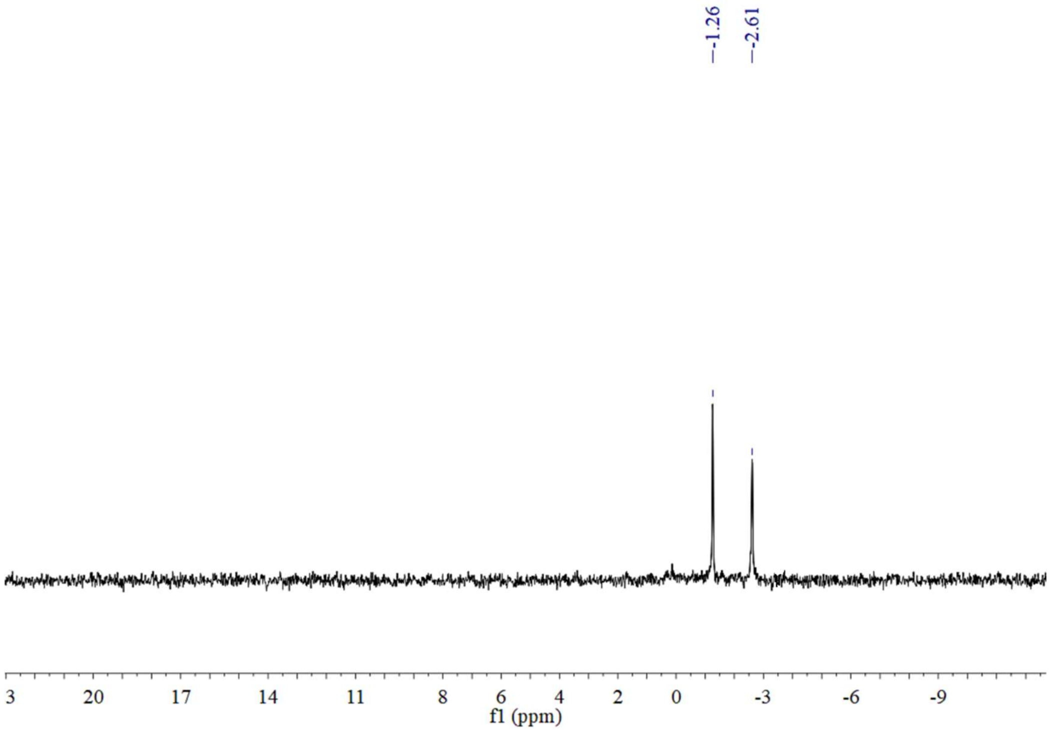
-1.26
-2.61
3
20
17
14
11
8
6
4
2
0
-3
-6
-9
f1 (ppm)

8b:

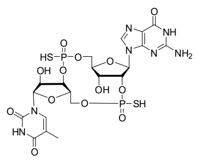
HS
OH
OH
NH
NH2
SH
HN

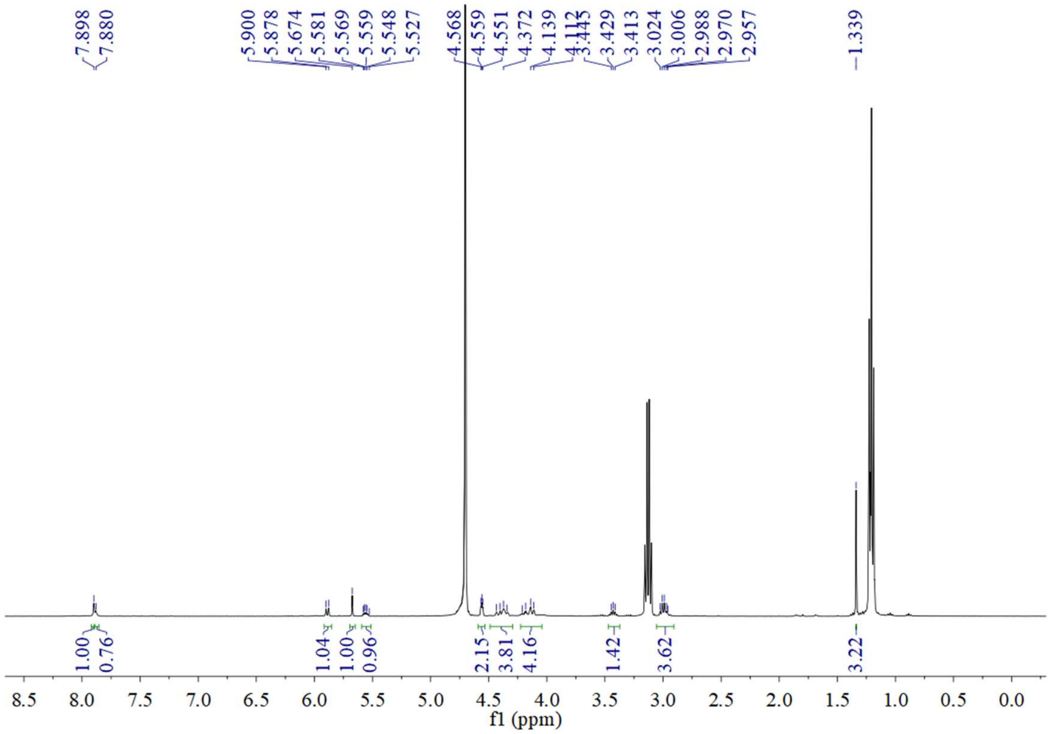
7.898
7.880
5.900
5.878
5.674
5.581
5.569
5.559
5.548
5.527
4.568
4.559
4.551
4.372
4.139
4.112
3.445
3.429
3.413
3.024
3.006
2.988
2.970
2.957
1.339
1.00
0.76
1.04
1.00
0.96
2.15
3.81
4.16
1.42
3.62
3.22
8.5 8.0 7.5 7.0 6.5 6.0 5.5 5.0 4.5 4.0 3.5 3.0 2.5 2.0 1.5 1.0 0.5 0.0
f1 (ppm)

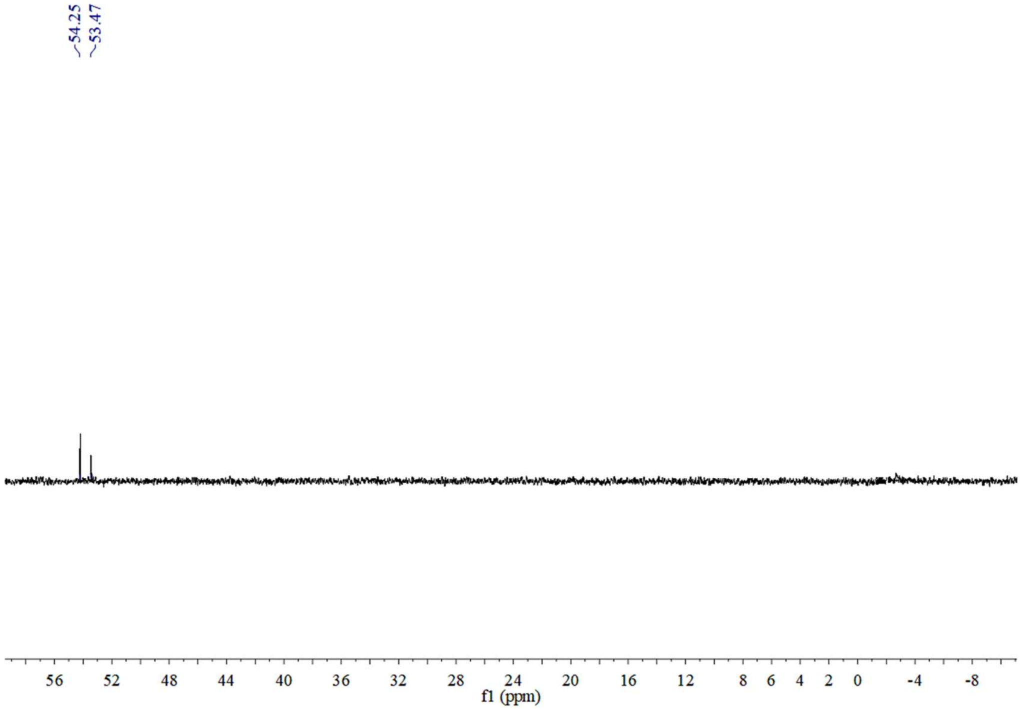
54.25
53.47
56 52 48 44 40 36 32 28 24 20 16 12 8 6 4 2 0 -4 -8
f1 (ppm)

8c:

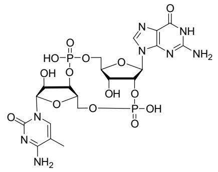

O
NH
NH2
HO
P
O
OH
O
N
OH
O
N
N
O
P
OH
NH2

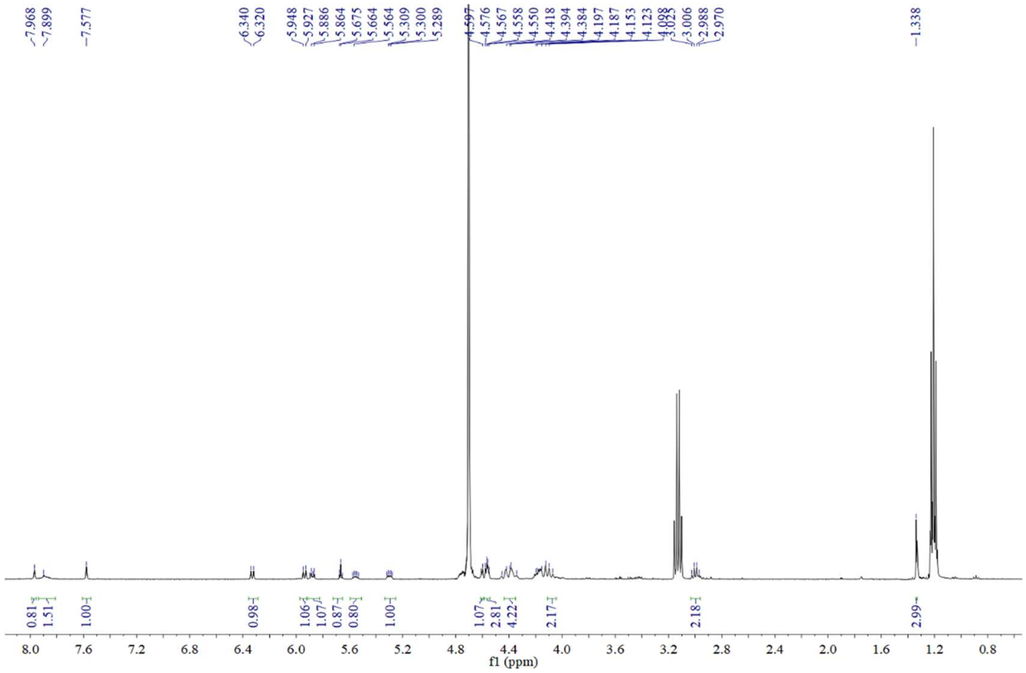

7.968
7.899
7.577
6.340
6.320
5.948
5.927
5.886
5.864
5.675
5.664
5.564
5.309
5.300
5.289
4.597
4.576
4.567
4.558
4.550
4.418
4.394
4.384
4.197
4.187
4.153
4.123
4.098
3.025
3.006
2.988
2.970
1.338
0.81
1.51
1.00
0.98
1.06
1.07
0.87
0.80
1.00
1.07
2.81
4.22
2.17
2.18
2.99
8.0 7.6 7.2 6.8 6.4 6.0 5.6 5.2 4.8 4.4 4.0 3.6 3.2 2.8 2.4 2.0 1.6 1.2 0.8
f1 (ppm)

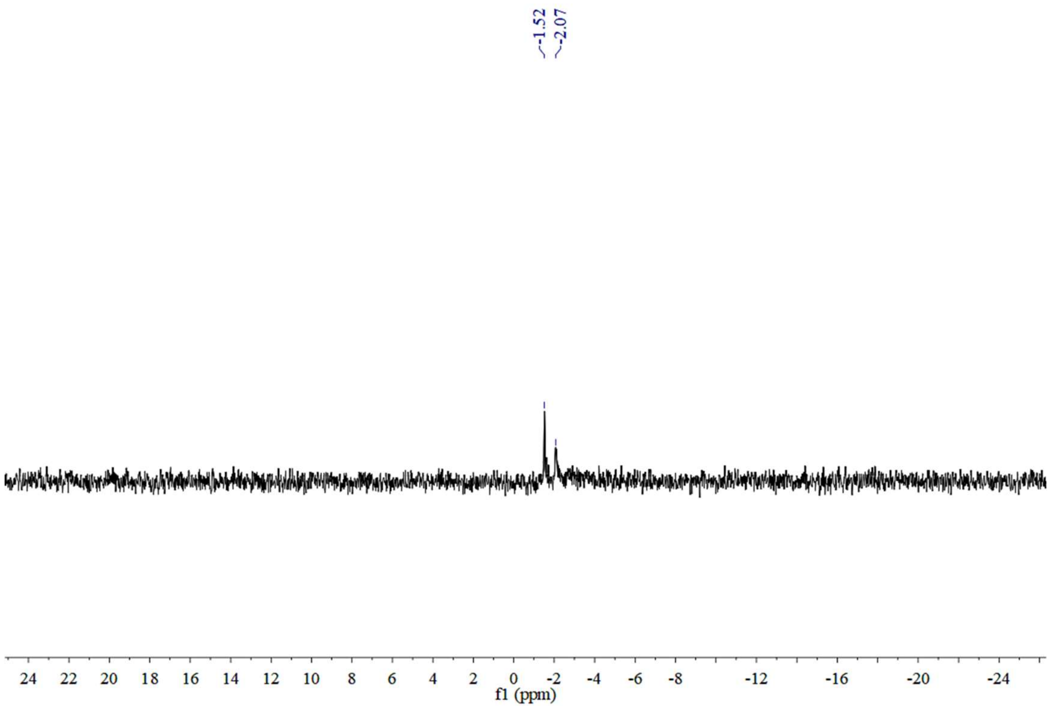

-1.52
-2.07
24 22 20 18 16 14 12 10 8 6 4 2 0 -2 -4 -6 -8 -12 -16 -20 -24
f1 (ppm)

8d:

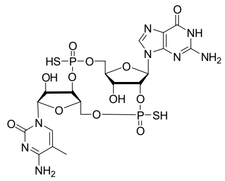

HS
O
OH
O
OH
N
NH
NH2
SH
NH2

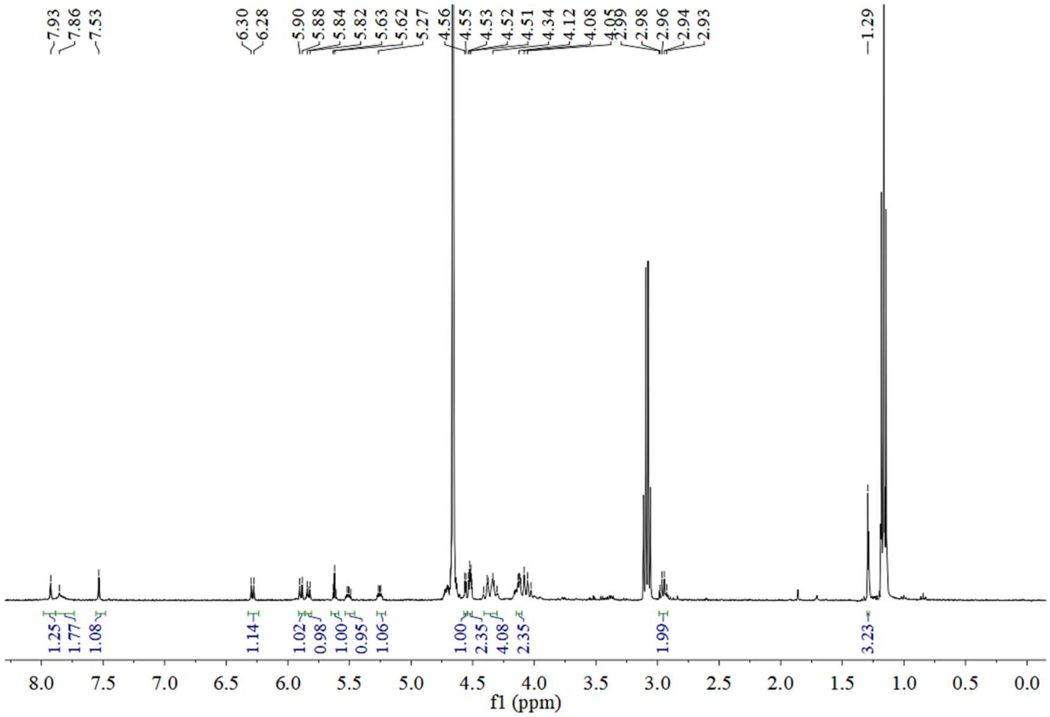

7.93
7.86
7.53
6.30
6.28
5.90
5.88
5.84
5.82
5.63
5.62
5.27
4.56
4.55
4.53
4.52
4.51
4.34
4.12
4.08
4.05
2.99
2.98
2.96
2.94
2.93
1.29
1.25
1.77
1.08
1.14
1.02
0.98
1.00
0.95
1.06
1.00
2.35
4.08
2.35
1.99
3.23
8.0 7.5 7.0 6.5 6.0 5.5 5.0 4.5 4.0 3.5 3.0 2.5 2.0 1.5 1.0 0.5 0.0
f1 (ppm)

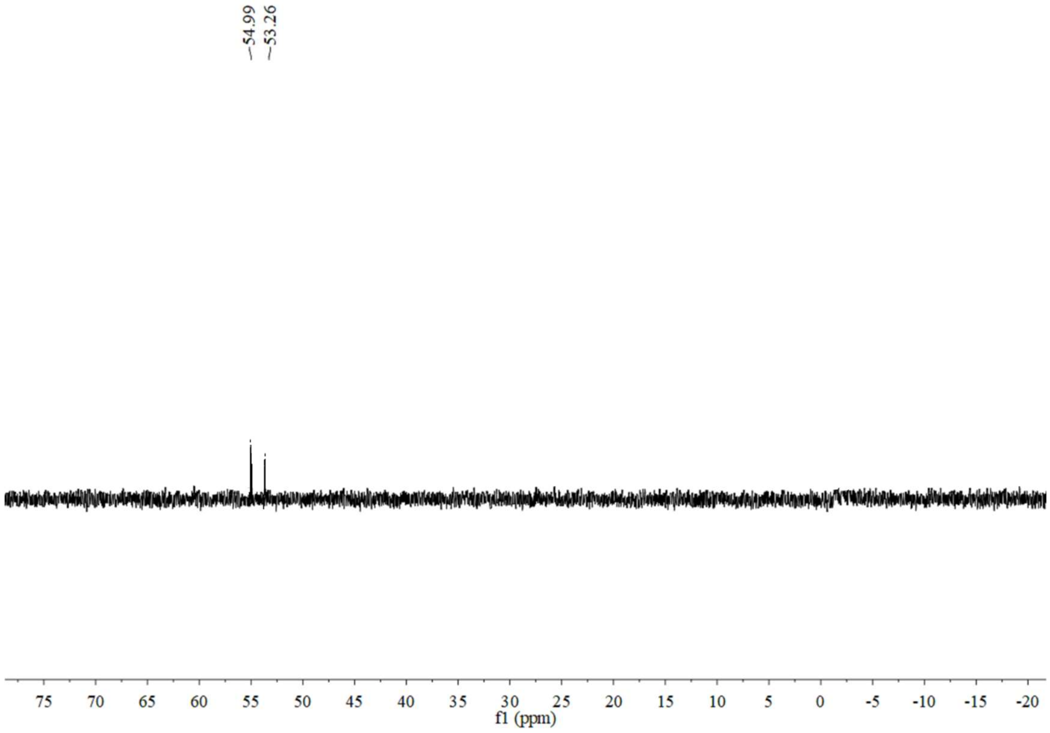

54.99
53.26
75 70 65 60 55 50 45 40 35 30 25 20 15 10 5 0 -5 -10 -15 -20
f1 (ppm)

12a:

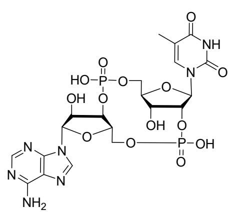

O
NH
O
N
HO
P
O
O
O
OH
O
O
OH
O
P
OH
O
N
N
N
N
NH$_2$

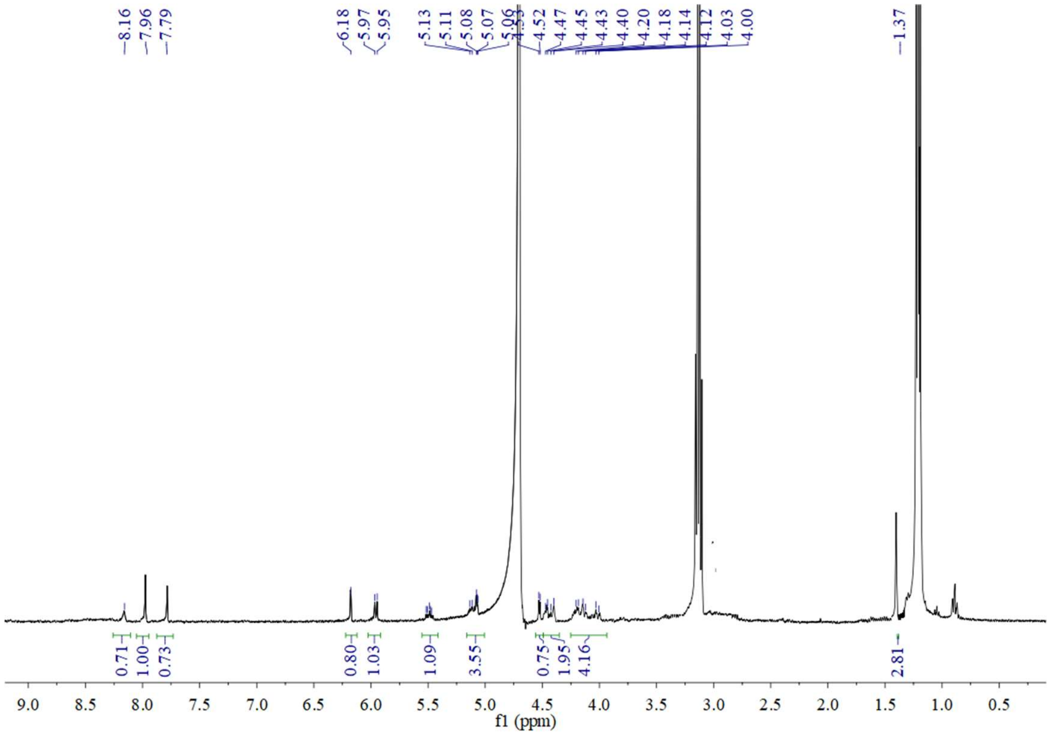

8.16
7.96
7.79
6.18
5.97
5.95
5.13
5.11
5.08
5.07
5.06
4.52
4.47
4.45
4.43
4.40
4.20
4.18
4.14
4.12
4.03
4.00
1.37
0.71
1.00
0.73
0.80
1.03
1.09
3.55
0.75
1.95
4.16
2.81
9.0 8.5 8.0 7.5 7.0 6.5 6.0 5.5 5.0 4.5 4.0 3.5 3.0 2.5 2.0 1.5 1.0 0.5
f1 (ppm)

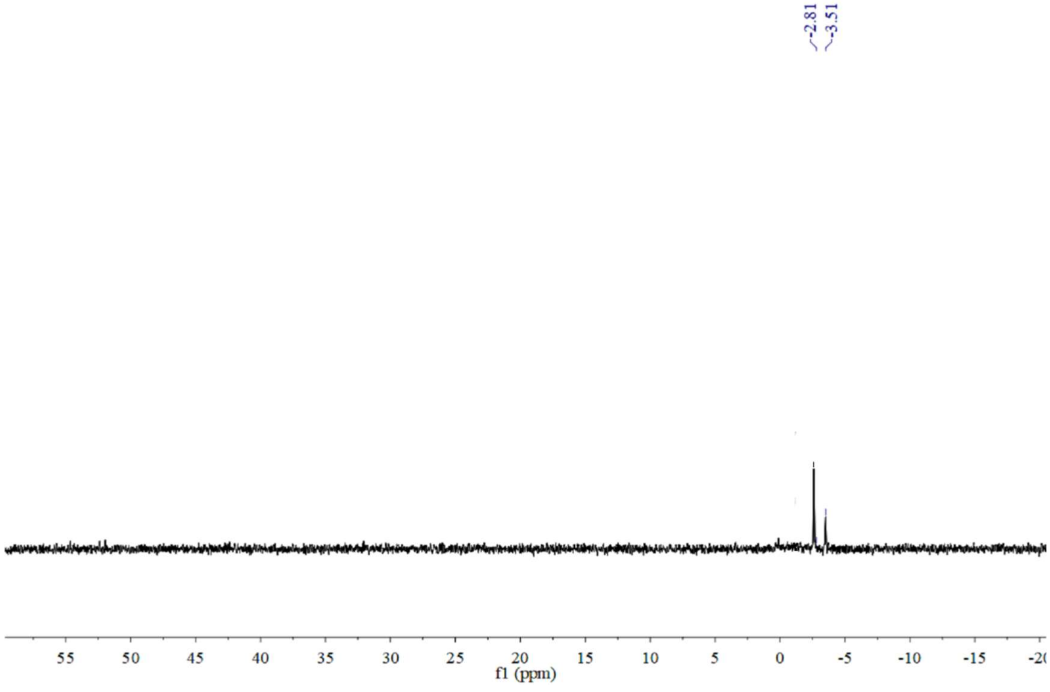

-2.81
-3.51
55 50 45 40 35 30 25 20 15 10 5 0 -5 -10 -15
f1 (ppm)

12b:

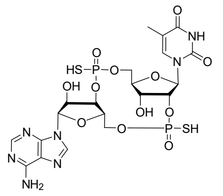

O
NH
O
N
HS
P
O
O
OH
O
O
OH
O
P
SH
O
N
N
N
N
NH2

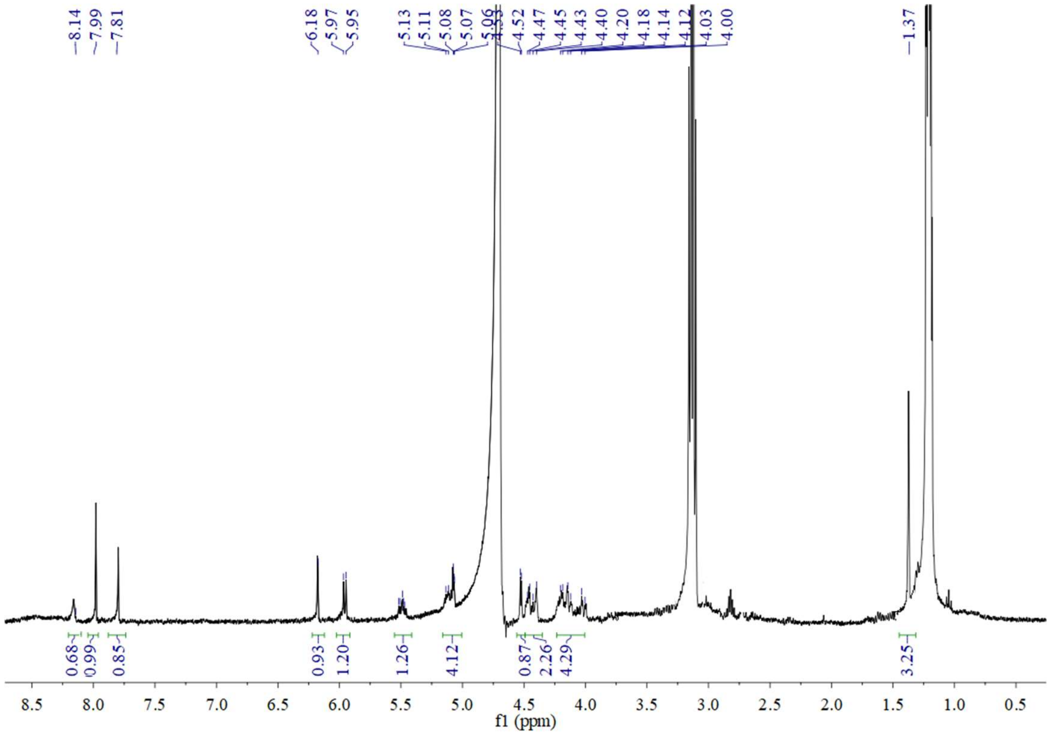

8.14
7.99
7.81
6.18
5.97
5.95
5.13
5.11
5.08
5.07
5.06
4.53
4.52
4.47
4.45
4.43
4.40
4.20
4.18
4.14
4.12
4.03
4.00
1.37
0.68
0.99
0.85
0.93
1.20
1.26
4.12
0.87
2.26
4.29
3.25
8.5
8.0
7.5
7.0
6.5
6.0
5.5
5.0
4.5
4.0
3.5
3.0
2.5
2.0
1.5
1.0
0.5
f1 (ppm)

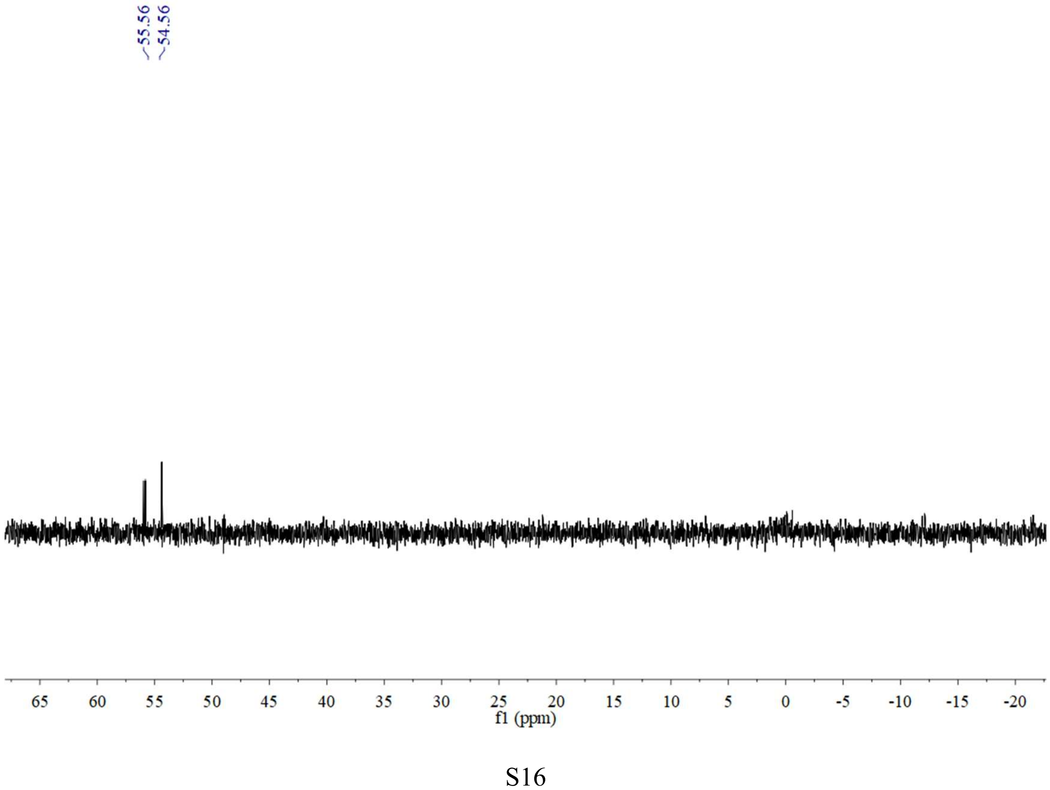

55.56
54.56
65
60
55
50
45
40
35
30
25
20
15
10
5
0
-5
-10
-15
-20
f1 (ppm)

12c:

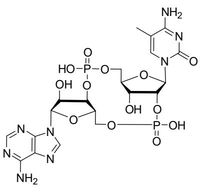

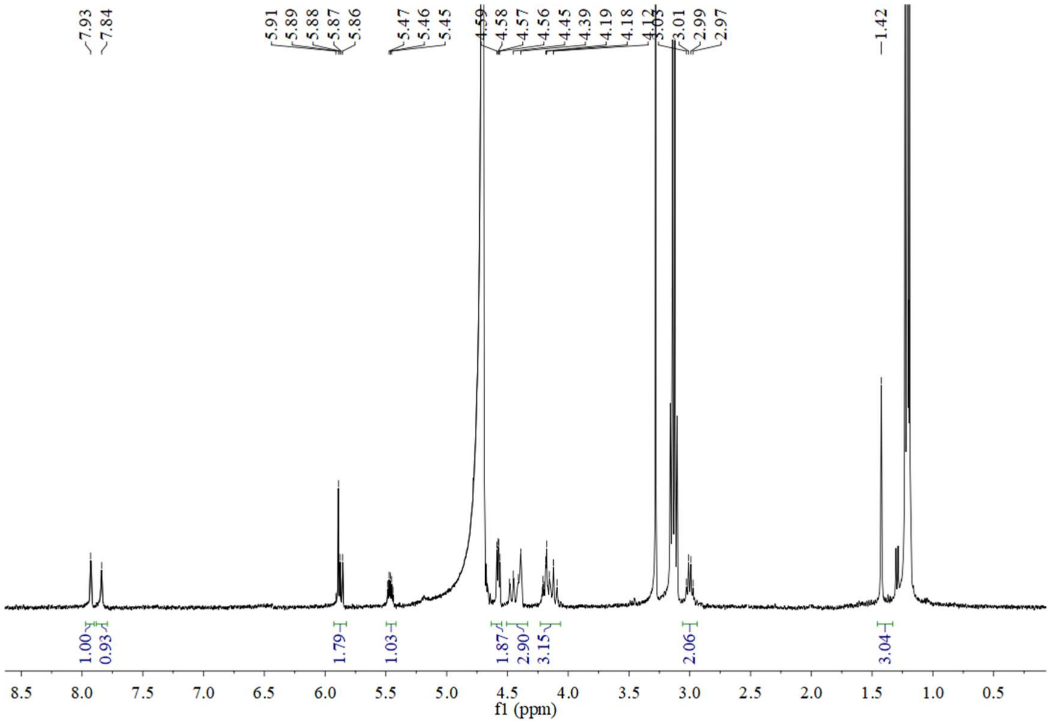
7.93
7.84
5.91
5.89
5.88
5.87
5.86
5.47
5.46
5.45
4.59
4.58
4.57
4.56
4.45
4.39
4.19
4.18
4.12
3.05
3.01
2.99
2.97
1.42
1.00
0.93
1.79
1.03
1.87
2.90
3.15
2.06
3.04
f1 (ppm)

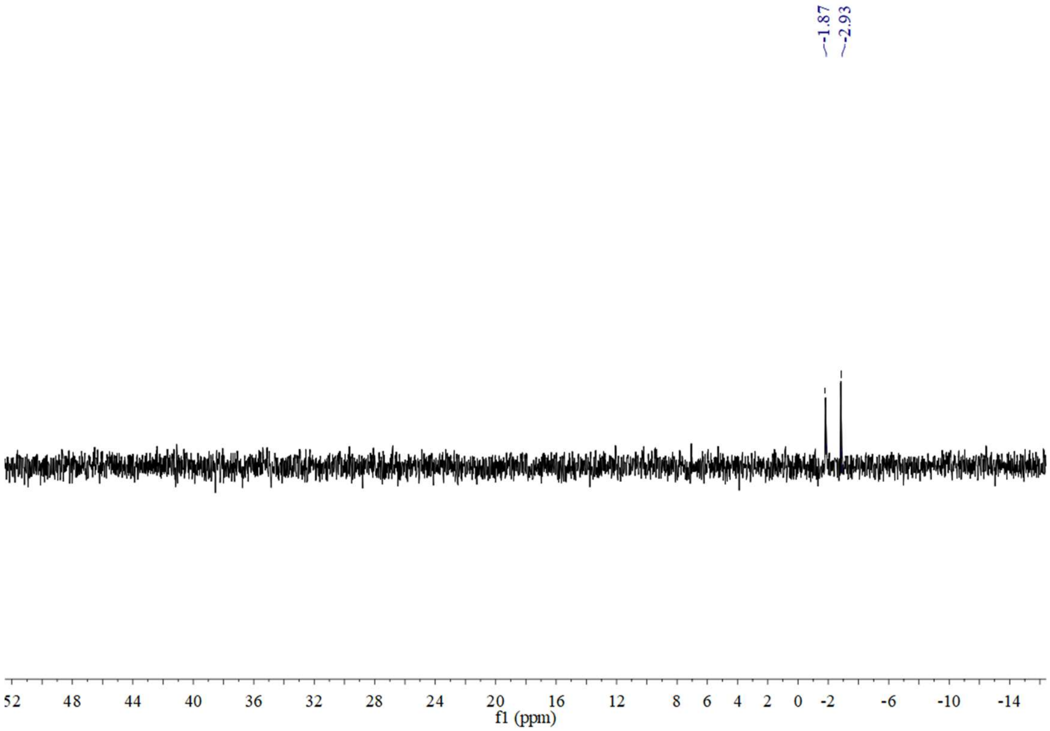
-1.87
-2.93
f1 (ppm)

12d:

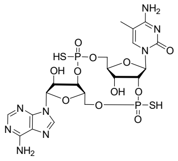
NH2
N
O
HS
P
O
OH
OH
SH
N
NH2

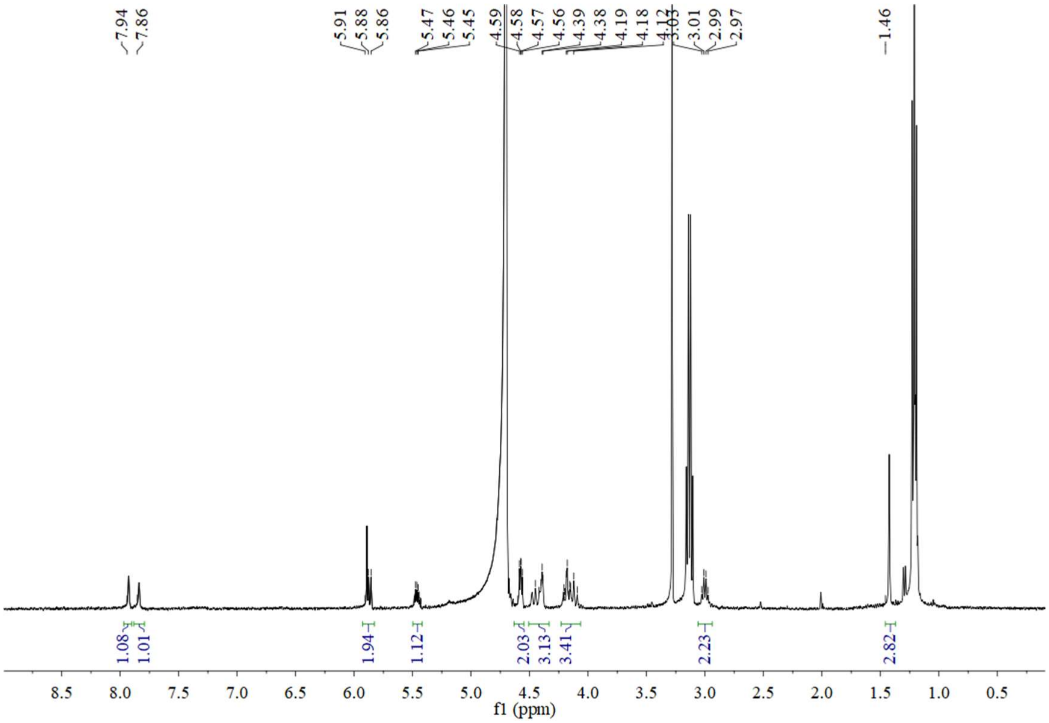
7.94
7.86
5.91
5.88
5.86
5.47
5.46
5.45
4.59
4.58
4.57
4.56
4.39
4.38
4.19
4.18
4.12
3.03
3.01
2.99
2.97
1.46
1.08
1.01
1.94
1.12
2.03
3.13
3.41
2.23
2.82
8.5 8.0 7.5 7.0 6.5 6.0 5.5 5.0 4.5 4.0 3.5 3.0 2.5 2.0 1.5 1.0 0.5
f1 (ppm)

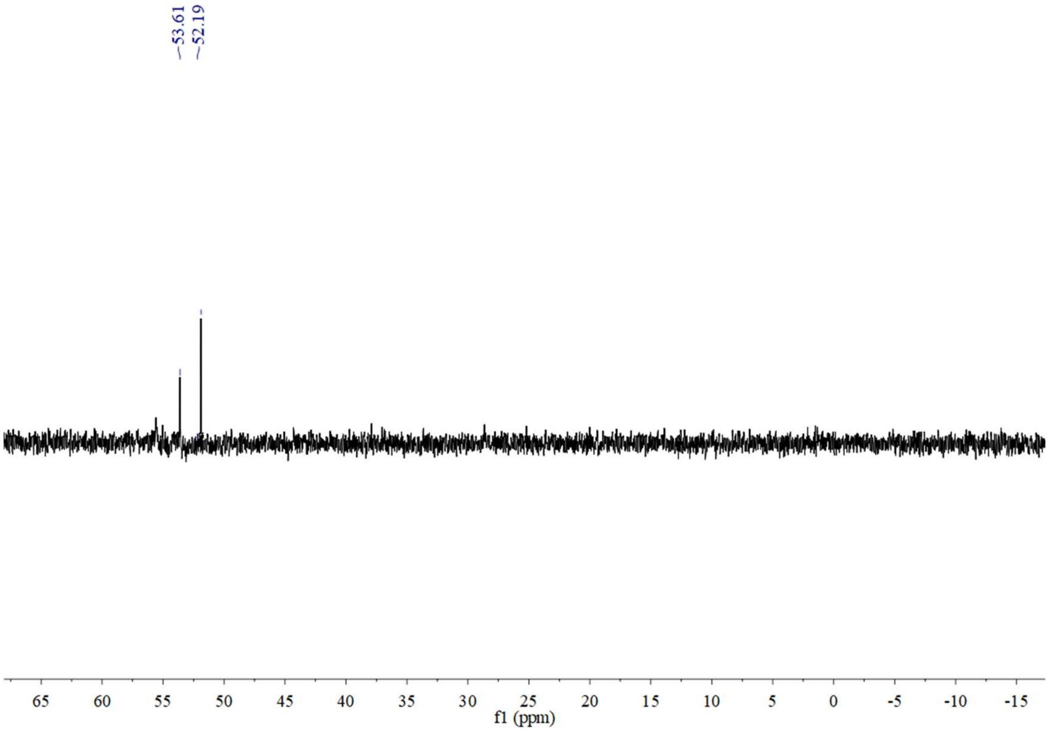
53.61
52.19
65 60 55 50 45 40 35 30 25 20 15 10 5 0 -5 -10 -15
f1 (ppm)
